# Phenotypic effects of A^m^ genomes in nascent synthetic hexaploids derived from interspecific crosses between durum and wild einkorn wheat

**DOI:** 10.1371/journal.pone.0284408

**Published:** 2023-04-27

**Authors:** Asami Michikawa, Moeko Okada, Tatsuya M. Ikeda, Kiyotaka Nagaki, Kentaro Yoshida, Shigeo Takumi

**Affiliations:** 1 Graduate School of Agricultural Science, Kobe University, Kobe, Hyogo, Japan; 2 Western Region Agricultural Research Center, National Agriculture and Food Research Organization, Fukuyama, Hiroshima, Japan; 3 Institute of Plant Science and Resources, Okayama University, Kurashiki, Okayama, Japan; 4 Graduate School of Agriculture, Kyoto University, Kyoto, Kyoto, Japan; Sichuan Agricultural University at Chengdu, CHINA

## Abstract

Allopolyploid speciation is a major evolutionary process in wheat (*Triticum* spp.) and the related *Aegilops* species. The generation of synthetic polyploids by interspecific crosses artificially reproduces the allopolyploidization of wheat and its relatives. These synthetic polyploids allow breeders to introduce agriculturally important traits into durum and common wheat cultivars. This study aimed to evaluate the genetic and phenotypic diversity in wild einkorn *Triticum monococcum* ssp. *aegilopoides* (Link) Thell., to generate a set of synthetic hexaploid lines containing the various A^m^ genomes from wild einkorn, and to reveal their trait characteristics. We examined the genetic diversity of 43 wild einkorn accessions using simple sequence repeat markers covering all the chromosomes and revealed two genetically divergent lineages, L1 and L2. The genetic divergence between these lineages was linked to their phenotypic divergence and their habitats. L1 accessions were characterized by early flowering, fewer spikelets, and large spikelets compared to L2 accessions. These trait differences could have resulted from adaptation to their different habitats. We then developed 42 synthetic hexaploids containing the AABBA^m^A^m^ genome through interspecific crosses between *T*. *turgidum* cv. Langdon (AABB genome) as the female parent and the wild einkorn accessions (A^m^A^m^ genome) as the male parents. Two of the 42 AABBA^m^A^m^ synthetic hexaploids exhibited hybrid dwarfness. The phenotypic divergence between L1 and L2 accessions of wild einkorn, especially for days to flowering and spikelet-related traits, significantly reflected phenotypic differences in the synthetic hexaploids. The differences in plant height and internodes between the lineages were more distinct in the hexaploid backgrounds. Furthermore, the AABBA^m^A^m^ synthetic hexaploids had longer spikelets and grains, long awns, high plant heights, soft grains, and late flowering, which are distinct from other synthetic hexaploid wheat lines such as AABBDD. Utilization of various A^m^ genomes of wild einkorn resulted in wide phenotypic diversity in the AABBA^m^A^m^ synthetic hexaploids and provides promising new breeding materials for wheat.

## Introduction

Allopolyploid speciation is one of the major evolutionary processes in wheat and its relatives. For the emergence of common wheat (*Triticum aestivum* L., AABBDD genome), triploid hybrids with the ABD genome were descended from interspecific crosses between tetraploid wheat (*Triticum turgidum* L., AABB) and diploid wild wheat (*Aegilops tauschii* Coss., DD), and unreduced gametes were formed in pollen and egg cells of the ABD hybrids. This evolutionary process can be reproduced through artificial crosses, and the resulting allohexaploid plants are called synthetic wheat hexaploid lines [1]. Synthetic wheat hexaploids have been useful in the study of genetic and epigenetic modifications in chromosomes during allohexaploidization and common wheat speciation [2–5]. Synthetic wheat lines with the AABBDD genome have been used as bridges to introduce agriculturally important traits from *Ae*. *tauschii* into common wheat [6–8]. In addition to AABBDD synthetic hexaploids, various synthetic allopolyploid lines have been exploited among wheat and its wild relatives, and alien chromosome addition lines and introgression lines have been produced to study the effects of the added and introduced chromosomal regions of the wild relatives.

**Fig 1:**
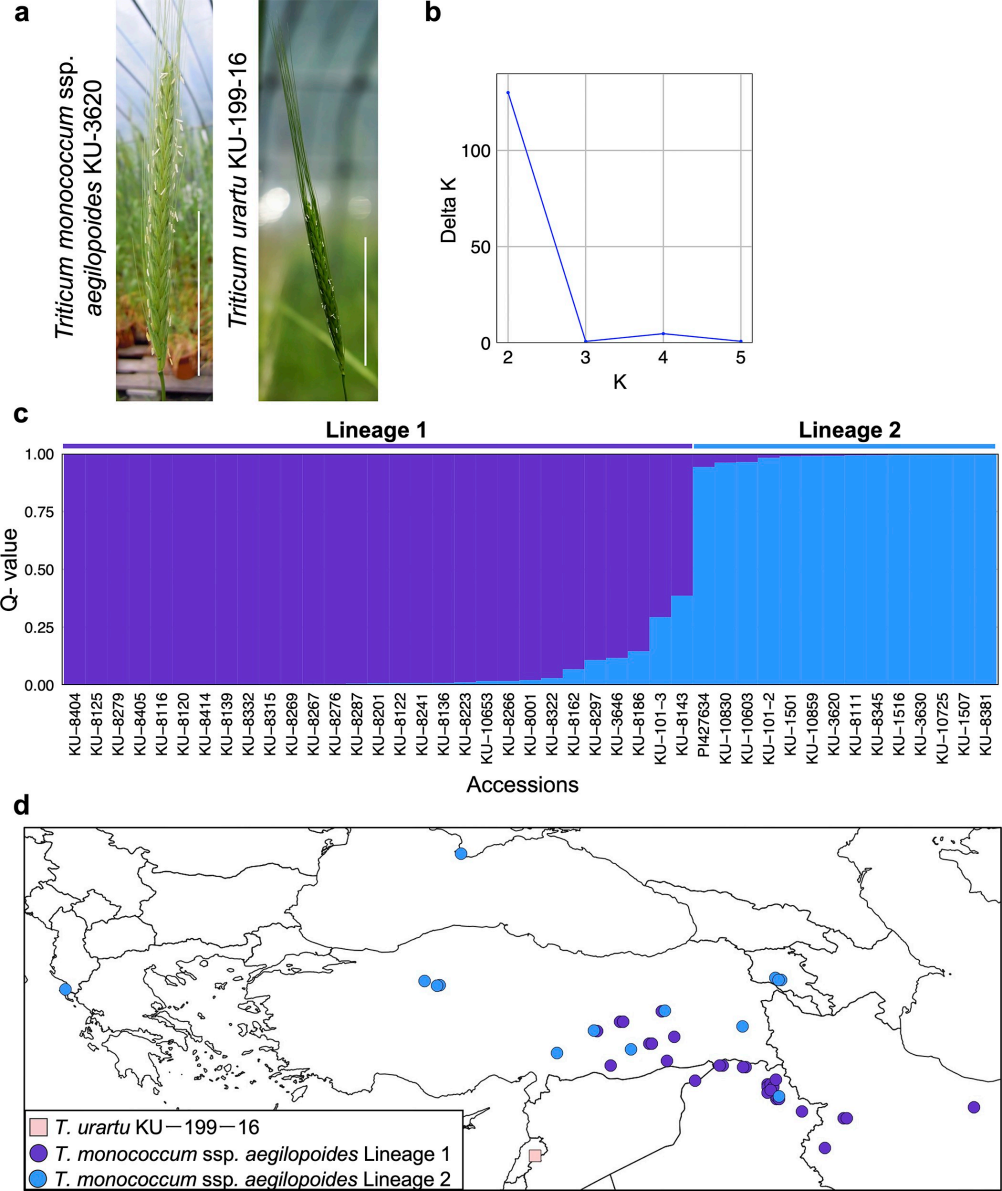
Subpopulation structure of *Triticum monococcum* ssp. *aegilopoides*. (a) Morphological differences of spikes between *T*. *monococcum* ssp. *aegilopoides* KU-3620 and *T*. *urartu* KU-199-16. White bars indicate 5 cm. (b) Estimation of the optimal number of sub-populations (k) from STRUCTURE with the ΔK method. (c) Proportion of membership of the 43 accessions for K = 2, as calculated by STRUCTURE and CLUMPP software based on the polymorphisms detected by SSR markers. (d) Geographic distribution of the lineage 1 (L1) and lineage 2 (L2) accessions of wild einkorn and one accession of *T*. *urartu*.

Einkorn wheat (*Triticum monococcum* L., A^m^A^m^) includes two subspecies: ssp. *monococcum* as the cultivated form and ssp. *aegilopoides* (Link) Thell. (syn. *T*. *boeoticum* Boiss) as the wild form. *Triticum urartu* Tumanian ex Gandilyan is the A-genome donor of tetraploid and common wheat [9] (Fig 1A). Interspecific hybrids between *T*. *monococcum* and *T*. *urartu* are almost sterile, and many chromosomal rearrangements occur between the A and A^m^ genomes [10,11], indicating genetic differentiation of the two genomes. Wild einkorn wheat, ssp. *aegilopoides*, displays wide genetic diversity [12–14] and is a valuable resource for disease resistance and grain quality-related characteristics in wheat breeding, as well as drought stress tolerance [15–22].

Synthetic hexaploid wheat lines containing the A^m^ genome have been produced and used for breeding. For example, AABBA^m^A^m^ synthetic hexaploids were produced from crosses between *T*. *turgidum* and *T*. *monococcum* ssp. *monococcum* [15,23–25] and the AAGGA^m^A^m^ synthetic hexaploids from crosses between *Triticum timopheevii* Zhuk. and *T*. *monococcum* ssp. *monococcum* [26,27]. Triploid F_1_ plants between tetraploid wheat and *T*. *monococcum* are sterile, whereas the AABBA^m^A^m^ amphiploids are meiotically stable and fully fertile [23]. Recently, an A^m^ genome-specific single nucleotide polymorphism (SNP) marker set was developed to characterize the introduced chromosomal segments of *T*. *monococcum* ssp. *monococcum* in common wheat [28]. Expression of the high-molecular-weight glutenin subunit gene *Glu-1* and stripe rust resistance from *T*. *monococcum* ssp. *monococcum* was successfully confirmed in the AABBA^m^A^m^ synthetic hexaploids [15,16]. Two powdery mildew resistance genes were transmitted to common wheat through a cross between the F_1_ progeny (ABA^m^) of tetraploid wheat and *T*. *monococcum* ssp. *aegilopoides* with common wheat [29]. A stripe rust resistant quantitative trait locus (QTL) was also transferred from *T*. *monococcum* ssp. *aegilopoides* to common wheat using tetraploid wheat as a bridging species [19]. Therefore, AABBA^m^A^m^ synthetic hexaploids are expected to be useful as bridges to introduce desirable genes into durum and common wheat cultivars.

The durum wheat cultivar Langdon (Ldn) is an efficient female parent used to produce synthetic hexaploid wheat lines with the AABBDD genome [1], and many synthetic hexaploids have been developed through crosses between Ldn and diverse *Ae*. *tauschii* accessions [30,31]. AABBDD synthetic hexaploids are useful for evaluating the effects of D-genome diversity in a hexaploid background. Synthetic hexaploid lines were recently developed through crosses of Ldn and various accessions of *Aegilops umbellulata* Zhuk. [32,33]. AABBUU synthetic hexaploids are expected to enlarge the diversity of grain hardness, heading/flowering date, and spike/spikelet morphology in common wheat. In this study, we generated a set of synthetic hexaploid lines with the various A^m^ genomes from accessions of *T*. *monococcum* ssp. *aegilopoides*. Nascent AABBA^m^A^m^ synthetic hexaploids should be differentially affected by the different A^m^ genome of each ssp. *aegilopoides* accession. First, we evaluated genetic diversity in 43 wild einkorn accessions. Then, we generated AABBA^m^A^m^ synthetic hexaploids using the 43 wild einkorn accessions as the pollen parents and Ldn as the female parent. We examined phenotypic variations in the synthetic hexaploids and their parental einkorn accessions to evaluate the transmission of traits. Finally, we compared phenotypes between the AABBA^m^A^m^ synthetic hexaploids and previously developed synthetic hexaploids that share the common tetraploid wheat female parent Ldn and revealed unique characteristics of the AABBA^m^A^m^ synthetic hexaploids.

## Materials and methods

### Plant materials

In this study, 43 accessions of *Triticum monococcum* ssp. *aegilopoides* (Link) Thell., an accession (KU-199-16) of *T*. *urartu* Tumanian ex Gandilyan, and the tetraploid wheat (*T*. *turgidum* L. ssp. *durum*) cultivar Langdon (Ldn) were used. The wild einkorn and *T*. *urartu* accessions are listed in S1 Table. These seeds, except for PI427634, were supplied by the National BioResource Project (NBRP)-Wheat with support in part by the National BioResource Project of the Ministry of Education, Culture, Sports, Science and Technology, Japan (https://shigen.nig.ac.jp/wheat/komugi/).

After removing anthers from the immature florets of Ldn, pollen of each of the diploid wheat accessions was crossed to Ldn as the female parent. Seedlings of the F_1_ hybrids were treated with 0.1% colchicine (Wako, Osaka, Japan) for 5 h and grown to maturity in a glasshouse at Kobe University (34°43’N, 135°13’E) to obtain the selfed seeds (F_2_ generation). Thus, the synthetic AABBA^m^A^m^ lines share the A and B genomes from Ldn and contain the A^m^ genome derived from diverse *T*. *monococcum* ssp. *aegilopoides* accessions. The somatic chromosome numbers were determined from root-tip mitotic preparations of the F_3_ plants using the standard acetocarmine squash method. Four lines of synthetic AABBDD hexaploids, Ldn/KU-2097 (Syn6214), Ldn/IG126387 (Syn6240), Ldn/PI476874 (Syn6256), and Ldn/KU-2069 (Syn6262) [34], were also used and grown under the same conditions as the AABBA^m^A^m^ hexaploids.

### SSR-based PCR analysis of the wild einkorn parental accessions

Forty-two SSR markers covering all the chromosomes were selected based on the linkage maps of the A genome [35,36]. The primer sequences and the respective annealing temperatures of the SSR markers were obtained from the NBRP KOMUGI website (http://www.shigen.nig.ac.jp/wheat/komugi/strains/aboutNbrpMarker.jsp) and the GrainGenes website (http://wheat.pw.usda.gov/GG2/maps.shtml). The SSR markers used in this study are listed in S2 Table. For SSR genotyping, 40 cycles of PCR were performed using 2× Quick Taq HS DyeMix (TOYOBO, Osaka, Japan) under the following conditions: 20 s at 94°C, 30 s at the annealing temperature, and 30 s at 68°C. PCR products were resolved in 2% agarose or 15% non-denaturing polyacrylamide gels, stained with ethidium bromide, and visualized under UV light according to our previous report [37]. To quantify genetic diversity in wild einkorn, Simpson’s index, expected heterozygosity, evenness, and genetic accumulation curves for the SSR markers were calculated using the R package’s Poppr version 2.8.3 [38] and ape version 5.3 [39].

The population structure and proportion of membership (Q) for the wild einkorn accessions were inferred using STRUCTURE v2.2.3 software [40]. The STRUCTURE analysis was conducted using the admixture model without prior population information of the wild einkorn accessions. A number of populations (*K*) ranging from 1 to 5 were examined. Ten runs were performed for each K with a burn-in period of 0.5 × 10^6^ iterations followed by 1 × 10^6^ iterations of Markov chain Monte Carlo methods. The ΔK [41] was estimated using Structure Harvester [42] to infer the optimal K value. CLUMPP version 1.1.2 [43] was used to obtain the summation of the Q-matrices deduced by the 10 independent runs of STRUCTURE under the optimal K value. The wild einkorn accessions were sorted into lineages based on the most probable Q-values.

### Genomic *in situ* hybridization (GISH)

GISH analysis of mitotic metaphase chromosomes was performed according to a previously described fluorescence *in situ* hybridization protocol with minor modifications[33,44]. Genomic DNA was extracted from young leaves of a *T*. *monococcum* ssp. *aegilopoides* accession, KU-3620. Genomic DNA of KU-3620 was labeled using Biotin-Nick Translation Mix (Roche Diagnostics, Basel, Switzerland), incubated at 16°C for 24 h, and then digested with the restriction enzyme *Hae*III at 37°C for 5 h. The biotin-labeled DNA was used as a probe. After chromosomes were incubated in 2× SSC (saline-sodium citrate) buffer including 70% (v/v) formamide at 80°C for 2 min for denaturation, they were hybridized with the biotin-labeled probe. The biotin-labeled probe was visualized using streptavidin-conjugated Alexa Fluor 555 (Life Technologies, Carlsbad, CA, USA). Chromosomes were counterstained with 0.1 μg/ml 4,6-diamino-2-phenylindole (DAPI). GISH signals and DAPI-stained chromosomes were captured using a fluorescence microscope (Axioskop2, Carl Zeiss, Oberkochen, Germany), and images were pseudo-colored and processed using ZEN software blue edition (Carl Zeiss).

### PCR analysis of the wild einkorn accessions and the AABBA^m^A^m^ synthetic hexaploids

For validation of the added A^m^ chromosomes in the synthetic hexaploids, PCR analysis with the A^m^-genome-specific marker of each A^m^ chromosome was conducted according to our previous study [45]. The A^m^-genome-specific markers were cleaved amplified polymorphic sequences (CAPS) converted from RNA sequencing-derived SNP information. Total DNA was extracted from leaves of the parental accessions and synthetic hexaploid lines. PCR conditions for genotyping were described in our previous report [33]. Marker names, amplification conditions, and restriction enzymes of the A^m^-genome-specific markers developed in the present study are shown in Table 1. PCR products and their digests were resolved in 2% agarose or 15% nondenaturing polyacrylamide gels.

**Table 1:** The A^m^-chromosome-specific CAPS markers developed in this study.

Marker name	Chr.	Position (bp)	Forward and reverse primer sequences (5’ to 3’)	Restriction enzyme	Annealing temp. (°C)
1A-9	1A	301,133,756	CGCAATGCAGCTCCCGAAAA TGTGTGAGGGGATGCTCAGC	*Bst*PI	58
2A-32	2A	111,410,167	ACTGTAGCAGGATCGTCCGC CGCAGGCCAGCAGTGTAATC	*Hha*I	60
3A-9	3A	714,155,627	TCTCCAATGCAACTTTCACG CATTGGGGCTAAACCAGTTG	*Mbo*I	54
4A-55	4A	238,382,739	CTGGCAGTCGGAGTGGTACA GAAAGCTTTGCAGGCCCACT	*Dde*I	62
5A-169	5A	118,666,482	TTGAACTCGGGACCTCTCGC AGCCCCAGCGAAATGGATCA	*Sfa*NI	58
6A-51	6A	294,652,546	AGGAAGCGAAGTGGATGGGG CACGTCGACACCCAACTTGC	*Kpn*I	62
7A-20	7A	314,410,689	CGTGTATCTCGACGGCCCAT AGAAGGCAAGCGCGAGAAGA	*Sfa*NI	60

The position of each marker was deduced according to information for the chromosomal position of the SNP site distinguished between the A and A^m^ genomes (Michikawa et al. 2019) [45].

### Phenotype measurement

To evaluate phenotypic traits of the synthetic hexaploids, four F_2_ and F_3_ seeds of each synthetic hexaploid line were sown in November 2017 and 2018, respectively. These F_2_ and F_3_ plants were grown in seasons 2017–2018 and 2018–2019 using clay pots arranged randomly in the glasshouse of Kobe University in which the temperature was not regulated. Two or three individuals per parental wild einkorn wheat accession were also grown in seasons 2017–2018 and 2018–2019 using clay pots in a vinyl house of Kobe University in which the temperature was not regulated. For the preparation of soil to the pots, a mixture of fertilizers containing 8% each of N, P, and K were applied to soil at approximately 70 g/m^2^. Eighteen traits: heading time, flowering time, flag leaf length, flag leaf width, top awn length, middle awn length, bottom awn length, first internode length, second internode length, third internode length, fourth internode length, fifth internode length, plant height, stem width, spike length, the number of spikelets, spikelet length, and spikelet width were tested. The traits were evaluated in three replications per individual. Heading time and flowering time were the number of days from sowing to heading and flowering, respectively. Stem length corresponded to the length from the crown to the neck of the spike. Plant height was the sum of stem length and spike length. Stem width was measured at the middle of the 1^st^ internode. Spikelet length and spikelet width were measured for three spikelets per spike.

The size and shape of grains harvested in 2018–2019 were measured in each synthetic hexaploid line using *SmartGrain* software ver. 1.2, which was developed for high-throughput phenotyping of rice (*Oryza sativa*) seeds [46]. Six parameters for grain size and shape, including grain area size, grain perimeter length, grain length, grain width, grain length-width ratio, and grain circularity, were recorded for at least 50 seeds of each accession and line according to the *SmartGrain* protocol.

Four grain-related traits, grain hardness, weight, diameter, and moisture were evaluated using Single-Kernel Characterization System (SKCS) 4100 (Perten, Stockholm, Sweden). The SKCS hardness index was obtained from crushing a sample of at least 50 kernels from Ldn and each synthetic hexaploid, similar to our previous reports [32,33]. A transverse section of grain was observed by a scanning electron microscope (S-3400N, Hitachi High-Technology, Tokyo, Japan) after the grain was snapped in the middle as previously described [47]. SEM observation was conducted without any pretreatment at an accelerating voltage of 8.00 kV under low vacuum conditions of 70 Pa at −25°C, and these conditions were similar to our previous reports [32,47]. VideometerLab 4 (Analytik, Cambridge, UK) was used to collect information of seed color on three parameters: CIELab L*, a*, and b*. CIELab L* indicates lightness, CIELab a* indicates redness, and CIELab b* indicates yellowness.

### Statistical analysis

We analyzed the trait measurements using generalized linear mixed models (GLMM) implemented in Bayesian generalized (non-)linear multivariate multilevel models using the Stan (brms) package [48]. To evaluate the effect of lineage differences and seasonal differences on phenotypic traits in the synthetic hexaploids and their parent einkorn accessions, posterior distributions of means of explanatory variables were estimated under the model described in the following equation (Model A).


$$\gamma_{i}∼Normal(0,\delta_{i}^{2})$$

$$\mu_{i}=\beta_{0}+\beta_{1}x_{i1}+\beta_{2}x_{i2}+\gamma_{i}$$

$$y_{i}∼Normal(\mu_{i},\delta^{2})$$

Individual identification was incorporated into the model as random effect *γ*_*i*_. Lineage differences and seasonal differences were incorporated into the model as fixation effects *β*_1_ and *β*_2_. *β*_0_ is the intercept of the fixed effect. The measurements of traits were response variables *y*_*i*_. Significant differences in traits between the lineages and between seasons were evaluated based on 95% credible intervals computed from the posterior distributions. To examine the effect of lineage differences on the grain morphology, posterior distributions of means of explanatory variables were estimated using generalized linear model (GLM) implemented in brms. The model is described in the following equation (Model B).


$$\mu_{i}=\beta_{0}+\beta_{1}x_{i}$$

$$y_{i}∼Normal(\mu_{i},\delta^{2})$$

Intercept and lineage differences were explanatory variables as fixed effects *β*_0_ and *β*_1_, and the measurements of grain morphology were response variables *y*_*i*_. To test the effect of temperature after anthesis on grain filling, we also tested GLMM implemented in brms (Model C). Model C incorporated the period (days) from anthesis to reaching a thermal time of 600°C days as a random effect into Model A instead of individual identification. The thermal time of 600°C days was calculated based on the daily average temperature observed at the Kobe Meteorological Observatory. Although the duration of grain filling is dependent on the cultivar, 498°C days for *T*. *aestivum* cv. Apache and 567°C days for *T*. *aestivum* cv. Renan have been reported [49]. Based on this observation, 600°C days was chosen as a parameter for temperature that influences grain filling.

Statistical comparisons of the traits among Ldn and the AABBA^m^A^m^, AABBAA, and AABBDD synthetic hexaploids were also evaluated based on GLMM implemented in brms. The model is described in the following equation (Model D).


$$\gamma_{i}∼Normal(0,\delta_{i}^{2})$$

$$\mu_{i}=\beta_{0}+\beta_{1}x_{i1}+\beta_{2}x_{i2}+\beta_{3}x_{i3}+\beta_{4}x_{i3}+\gamma_{i}$$

$$y_{i}∼Normal(\mu_{i},\delta^{2})$$

The differences among synthetic hexaploids with AABBA^m^A^m^, AABBAA, and AABBDD and seasonal differences were treated as fixed effects *β*_1_, *β*_2_, *β*_3_, and *β*_4_. *β*_0_ is an intercept of a fixed effect. Individual identification was treated as random effect *γ*_*i*_.

The posterior distributions of parameters in each model were estimated using default priors of brms and four chains with 5000 iterations. In each chain, the first 1000 iterations were a burn-in period for calibrating the sample. A total of 16,000 posterior samples were obtained. We confirmed that Rhat, which is used as a convergence diagnostic for MCMC, was less than 1.1 in all parameters of the models, indicating that all parameters were convergent. Based on the posterior distributions, the means, standard errors, and two-sided 95% credible intervals of coefficients were estimated. Model evaluation of Model B and Model C for grain morphological traits was conducted based on WAIC.

Average temperature per month and average precipitation per month for each habitat of the tested accessions from 1970 to 2000 were estimated based on WorldClim global climate datasets version 2.1 using the R package “raster” [50].

## Results

### Population structure of the wild einkorn wheat accessions

First, we characterized the genetic diversity and traits of 43 wild einkorn wheat (*T*. *monococcum* ssp. *aegilopoides*) accessions. To clarify the intraspecific genetic diversity and population structure of wild einkorn, simple sequence repeat (SSR) marker-based PCR analysis was performed using the 43 wild einkorn accessions that were used for creating the synthetic hexaploids (S1 Table). One *T*. *urartu* accession was used as an outgroup species. The genotype accumulation curve plateaued at 15 SSR markers, indicating that these markers were enough to discriminate the tested accessions (S1 Fig). All the tested markers were polymorphic and had two to seven alleles (S3 Table). Mean Simpson’s index, expected heterozygosity, and evenness for the SSR markers were 0.4639, 0.4747, and 0.6993, respectively.

To estimate the population structure of the 43 wild einkorn accessions, we conducted Bayesian clustering analysis. The ΔK for differing numbers of subpopulations presumed two clusters (k = 2) to be optimal (Fig 1B and 1C). These two clusters corresponded to the two distinct lineages in a previous RNA-seq-based polymorphism analysis [45]. The lineage mainly distributed in southern Turkey, Iraq, and Iran was called Lineage 1 (L1), and the other lineage distributed in Turkey, Greece, and Armenia was called Lineage 2 (L2) (Fig 1D).

### Generation of synthetic wheat hexaploids with the A^m^ genome

**Fig 2:**
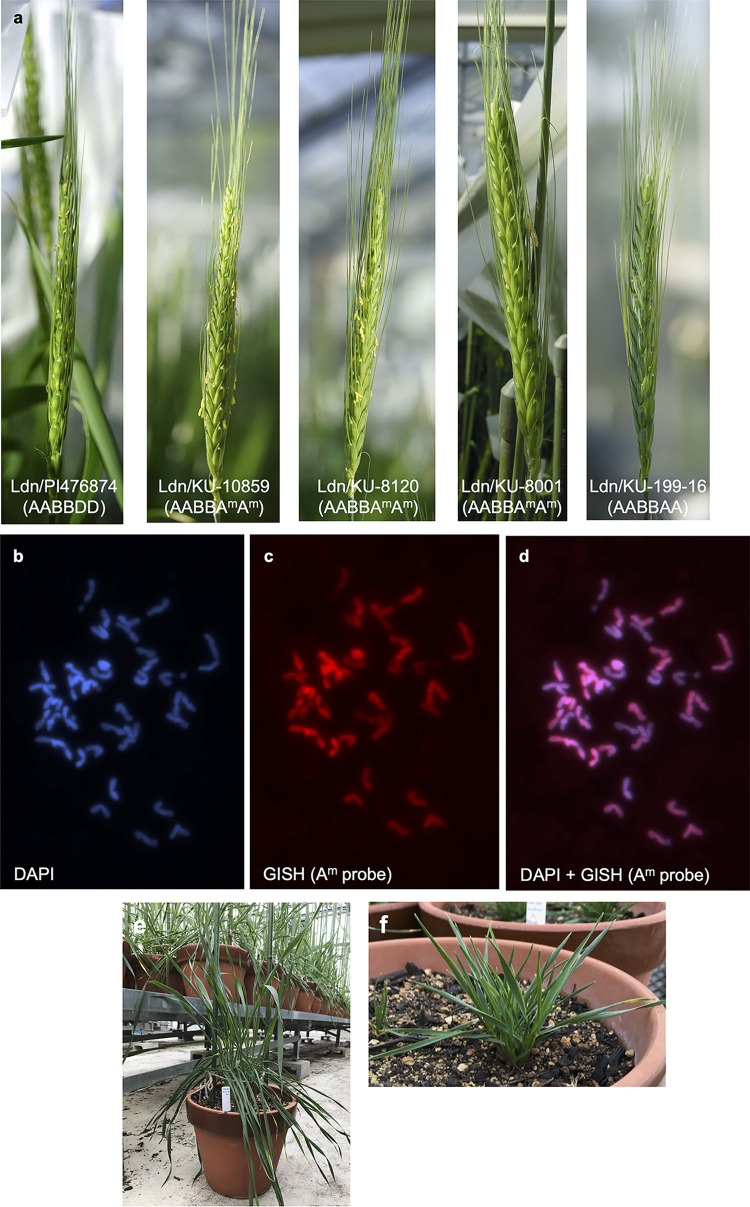
Spike morphology and cytological analysis of AABBA^m^A^m^ synthetic hexaploids. (a) Spikes of AABBDD, AABBA^m^A^m^, and AABBAA synthetic hexaploids. (b, c, d) GISH analysis of Ldn/*Triticum monococcum* ssp. *aegilopoides* KU-3620. Chromosomes were counterstained with DAPI (blue) (b), stained with an A^m^ (*T*. *monococcum* ssp. *aegilopoides* KU-3620 DNA, Alexa Fluor 555) genomic DNA probe for the A^m^ genome DNA (red) (c), and the images of DAPI-stained chromosomes and GISH signals were merged (d). (e) Wild-type plants. (f) Hybrid dwarf plants.

In total, 42 synthetic hexaploids were generated through interspecific crosses between Ldn and 42 accessions of *T*. *monococcum* ssp. *aegilopoides* (Fig 2A). These synthetic hexaploid lines produced self-pollinated seeds. We conducted GISH analysis to evaluate the somatic chromosomes of the AABBA^m^A^m^ synthetic hexaploids (Fig 2B–2D). Forty-two somatic chromosomes were observed in the root cells of the F_3_ plants as expected. GISH analysis using the wild einkorn DNA as probes detected 28 chromosomes of wild einkorn descent in the AABBA^m^A^m^ synthetic hexaploid. Fourteen chromosomes were stained with relatively stronger GISH signals among the 42 chromosomes of the AABBA^m^A^m^ polyploids, and the weaker-stained chromosomes were presumed to be the B-genome chromosomes. This observation indicated that the A^m^-genome chromosomes could not be clearly distinguished from the A-genome chromosomes by the GISH analysis using the wild einkorn DNA.

**Fig 3:**
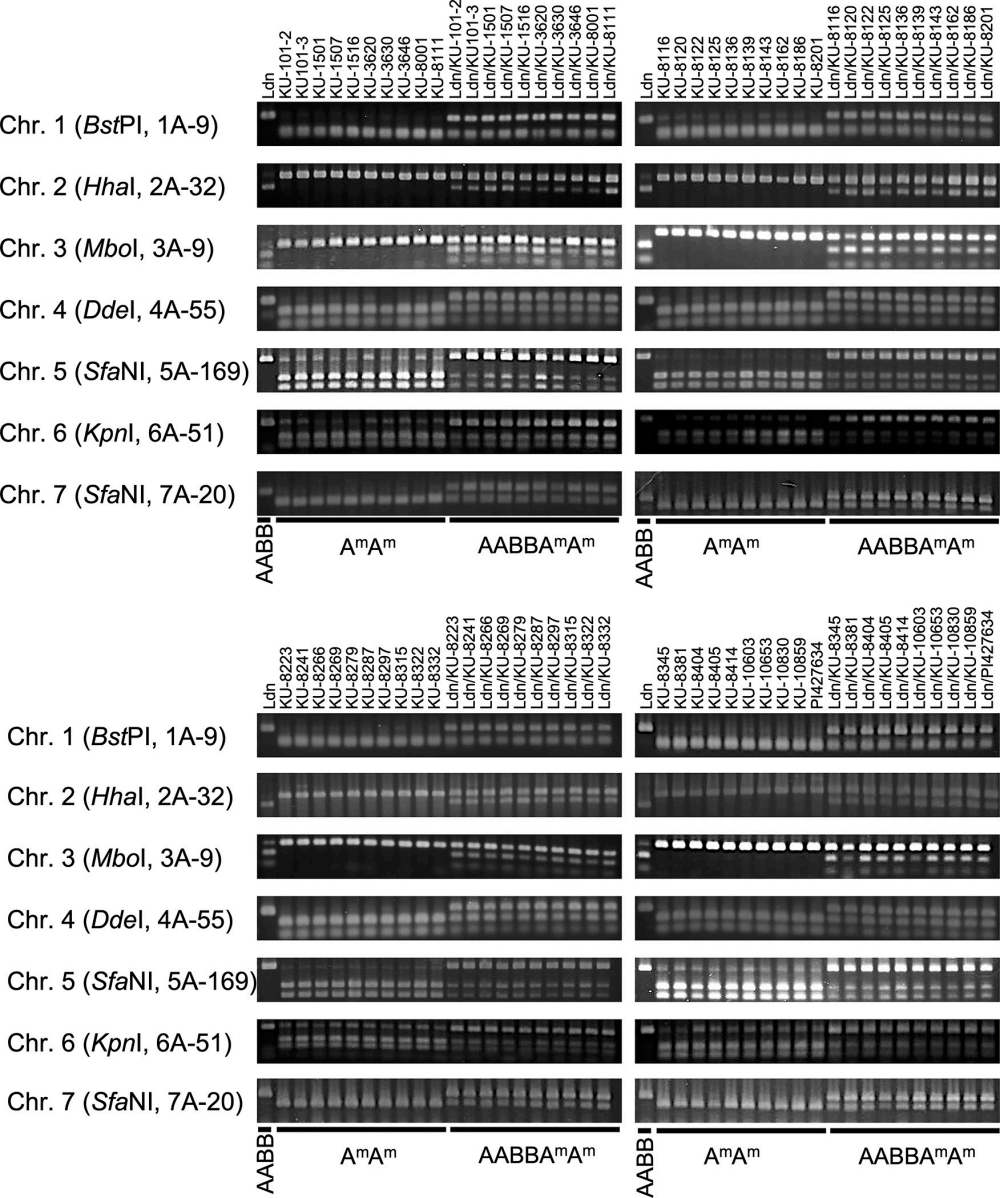
Confirmation of A^m^-genome chromosomes in the AABBA^m^A^m^ synthetic hexaploids. The presence of A^m^-genome chromosomes in the 40 AABBA^m^A^m^ synthetic hexaploid lines was confirmed based on the amplification of A^m^-chromosome-specific CAPS markers. Their parents (Ldn and wild einkorn accessions) were used as controls. Restriction enzyme and marker names are shown in parentheses following the chromosome names on the left of each gel image. Details of the CAPS markers are described in Table 1. Size differences in amplicons between the AB and A^m^ genomes were observed. Both amplicons from the AB and A^m^ genomes were detected in the synthetic hexaploid lines. The full-length gel images are shown in S2 Fig.

In nascent allohexaploid wheat, whole-chromosome aneuploidy has been reported [5]. To confirm the A^m^-genome chromosomes in the AABBA^m^A^m^ synthetics, we conducted PCR analysis with A^m^-chromosome-specific SSR markers (Figs 3 and S2, Table 1). The SSR markers used for this confirmation showed clear polymorphisms between Ldn and the wild einkorn accessions. For each of the A^m^-genome chromosomes, the AABBA^m^A^m^ synthetics contained both Ldn- and wild einkorn-derived PCR bands. Thus, all tested AABBA^m^A^m^ synthetic hexaploids contained a set of the A^m^-genome chromosomes. Two of the 42 AABBA^m^A^m^ synthetic hexaploids exhibited a hybrid dwarf (HDW) phenotype (Fig 2E and 2F). The two lines showing hybrid dwarfness contained the expected 42 chromosomes, including a set of the A^m^-genome chromosomes (S3 and S4 Figs). No visible necrosis was observed in the hybrid dwarf lines.

### Phenotypic characteristics of wild einkorn wheat accessions and their synthetics

**Fig 4:**
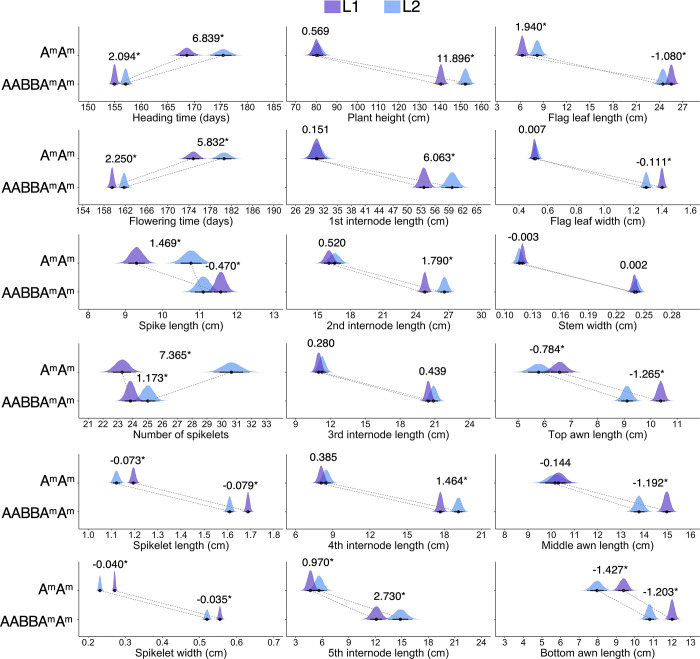
Phenotypic comparison between L1 and L2 of *Triticum monococcum* ssp. *aegilopoides* and the AABBA^m^A^m^ synthetic hexaploids. Posterior distributions of mean values for the 18 traits estimated under Bayesian GLMM. Violet and blue posterior distributions indicate mean values of L1 and L2, respectively. AABBA^m^A^m^ indicates the synthetic hexaploids and A^m^A^m^ indicates the einkorn accessions. The center point, thick line, and thin line below the posterior distribution designate mean, 80%, and 95% credible intervals, respectively. Dashed lines connect center points of each lineage between the synthetic hexaploids and the einkorn accessions. The difference between L1 and L2 (L2 − L1) is shown above the posterior distribution. Asterisks with the differences indicate that both the upper 95% credible interval and the lower 95% interval of the difference is above or below zero. Although the number of spikelets is discrete, a normal distribution was used as a probability distribution since a normal distribution was more supportive than a lognormal distribution and Poisson distribution in the model comparisons.

To estimate phenotypic variations in the 43 *T*. *monococcum* ssp. *aegilopoides* and their synthetics, we evaluated 18 traits in two seasons, 2017–2018 and 2018–2019 (S4 and S5 Tables). To characterize phenotypic variations in the wild einkorn accessions and their synthetics, we evaluated the effects of lineage differences and seasonal differences on their phenotypic variations based on Model A of Bayesian GLMM. The two HDW lines were excluded from the analysis because the hybrid dwarfness influenced their traits. The estimated means and 95% credible intervals of the coefficients of Model A are summarized in S6 and S7 Tables. The 95% credible intervals of the estimated means of 10 traits in the wild einkorn and 16 traits in the synthetic hexaploids were discriminated between the lineages (Fig 4). In the synthetic hexaploids, the plant height, internode length, flowering time, and heading time were longer in L2 than in L1. The number of spikelets in L2 was more than in L1. The awn length, spikelet length, and spikelet width were shorter in L2 than in L1. To varying degrees, these trait differences in the synthetic hexaploids corresponded to those in the wild einkorn. Furthermore, the lineage differences in the plant height and the internode length were more pronounced in the synthetic hexaploids than in the wild einkorn. However, differences in the flag leaf length and the spike length between the lineages were opposite between synthetic hexaploids and the wild einkorn. The flag leaf length and the spike length in L2 were shorter than in L1 in the wild einkorn, while those in L2 were longer than in L1 in the synthetic hexaploids.

**Fig 5:**
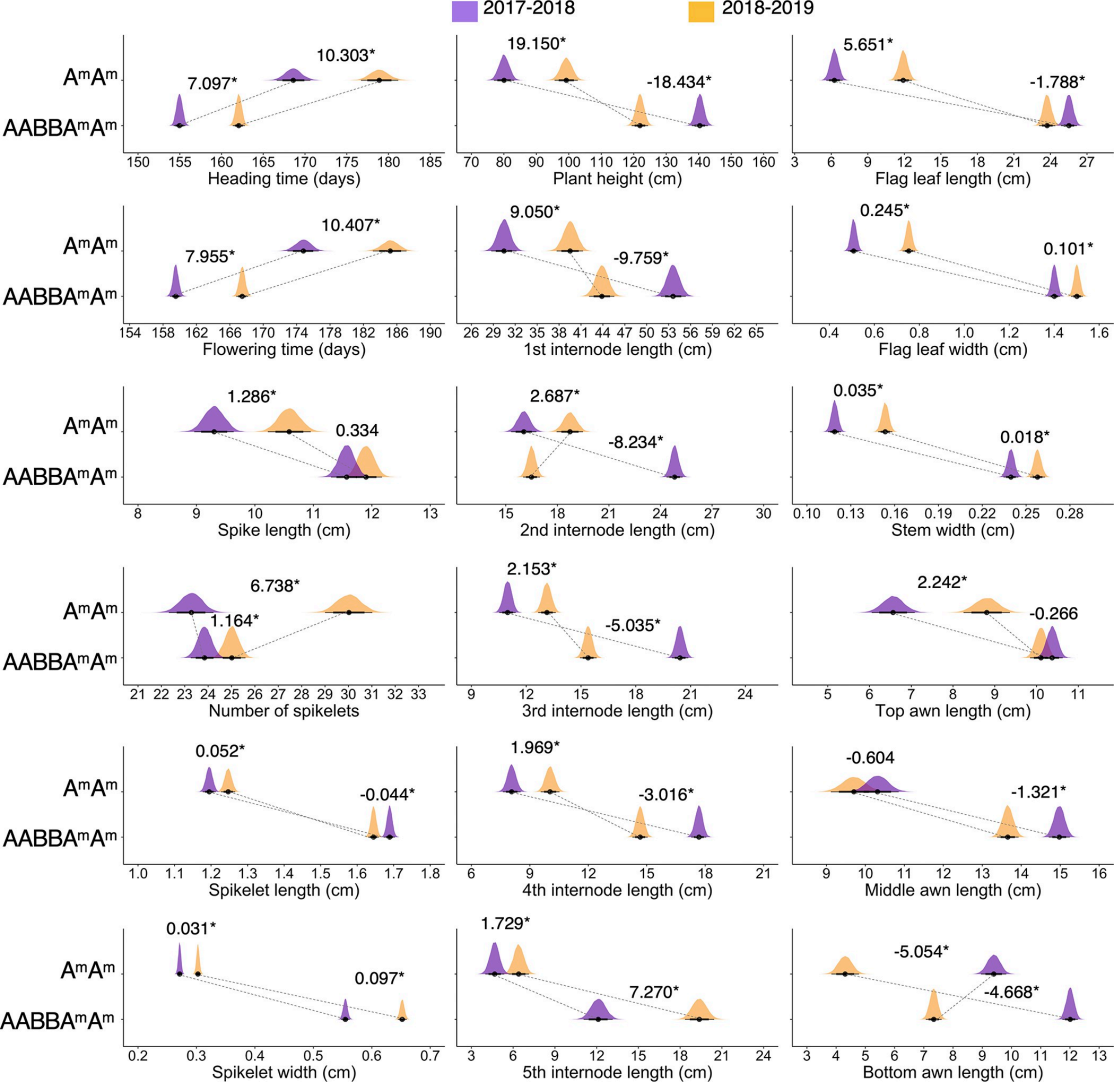
Phenotypic comparison between the 2017–2018 season and 2018–2019 season of *Triticum monococcum* ssp. *aegilopoides* and the AABBA^m^A^m^ synthetic hexaploids. Posterior distributions of mean values for the 18 traits estimated under Bayesian GLMM. Violet and orange posterior distributions indicate mean values of 2017–2018 and 2018–2019 seasons, respectively. AABBA^m^A^m^ indicates the synthetic hexaploids and A^m^A^m^ indicates their parental einkorn accessions. The center point, thick line, and thin line below the posterior distribution designate mean, 80%, and 95% credible intervals, respectively. Dashed lines connect center points of each season between the synthetic hexaploids and the einkorn accessions. Differences between the seasons are shown above the posterior distributions. Asterisks with the differences indicate that both the upper 95% credible interval and the lower 95% interval of the difference is above or below zero.

Seasonal differences were detected for all traits in the wild einkorn and synthetic hexaploids and were more prominent than the lineage differences (Fig 5). The extent of the interseasonal phenotypic difference was not at all reduced in the synthetic hexaploids. The differences in plant height and internode length between the seasons were increased in the synthetic backgrounds. The effects of the seasonal differences on plant height and internode length, except the 5th internode length and the spikelet length, were opposite between the wild einkorn and the synthetic hexaploids.

### Phenotypic characteristics of the synthetic hexaploid with hybrid dwarfness

To characterize the hybrid dwarfness in AABBA^m^A^m^ hexaploids, the 18 traits were compared between the two hybrid dwarf lines and the 40 normal height lines (S5 and S6 Figs, S5 Table). Heading time and flowering time of the hybrid dwarf lines were significantly delayed. All the internodes of the hybrid dwarf lines were significantly shorter than those of the normal height lines, resulting in significantly shorter plant height in the hybrid dwarf lines. The hybrid dwarf lines also showed shorter spikes, flag leaves, bottom awns, middle awns and spikelets and narrower flag leaves, stems and spikelets. Average self seed fertility of the normal height hexaploid lines was 92.3 ± 8.2%, whereas average self seed fertility of the hybrid dwarf lines dropped to 56.9 ± 12.23%.

### Characterization of grain morphology of the AABBA^m^A^m^ synthetic hexaploids

**Fig 6:**
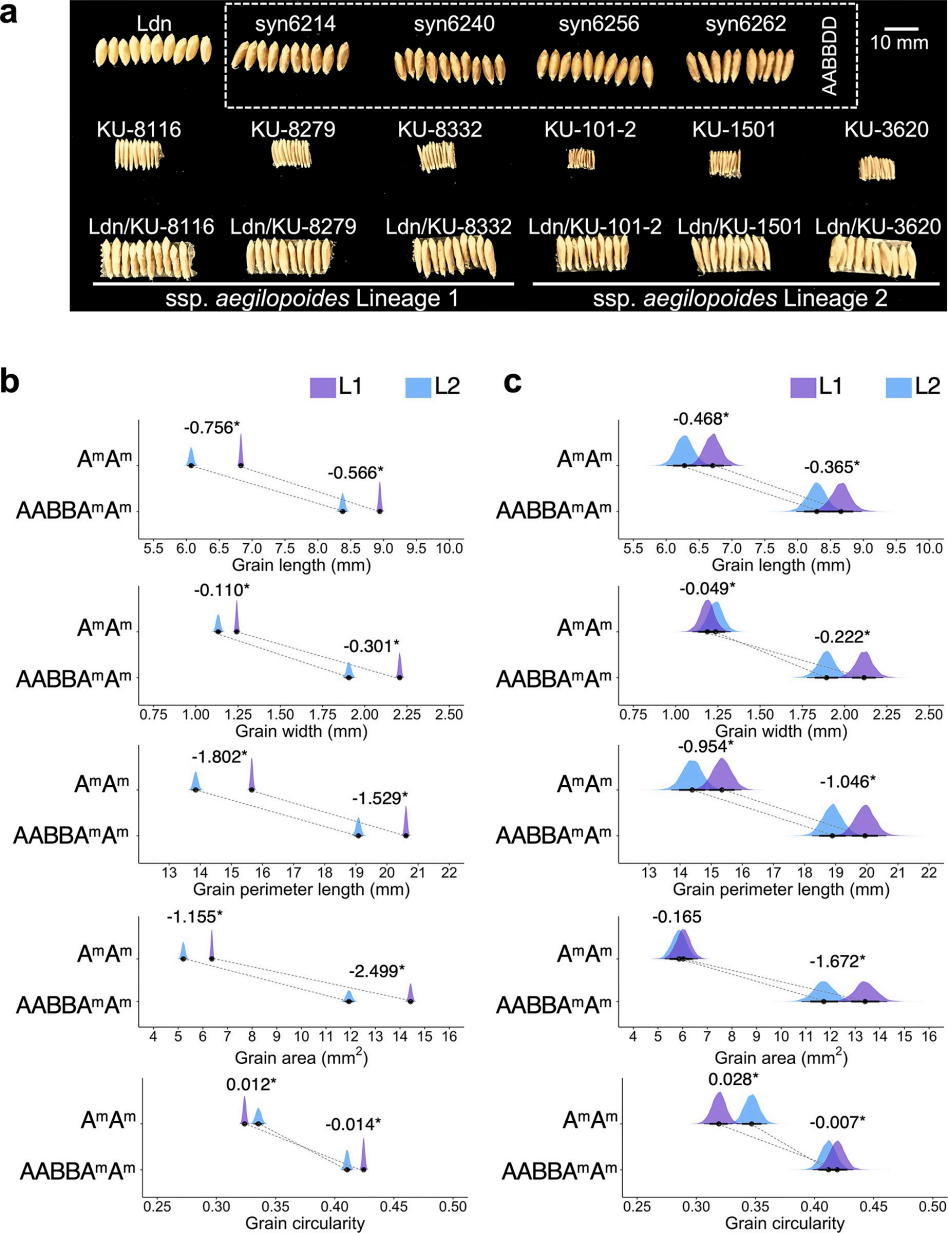
Comparisons of grain morphology between L1 and L2 of *Triticum monococcum* ssp. *aegilopoides* and the AABBA^m^A^m^ synthetic hexaploids. (a) Seed shapes among the AABBA^m^A^m^ synthetic hexaploids, their parental wild einkorn accessions, the AABBDD hexaploid lines, and Ldn are shown. (b) Posterior distributions of mean values for the five grain traits estimated under Bayesian GLM without thermal days as an explanatory variable. (c) Posterior distributions of mean values for the five grain traits estimated under Bayesian GLMM with thermal days. Violet and blue posterior distributions indicate mean values of L1 and L2, respectively. AABBA^m^A^m^ indicates the synthetic hexaploids and A^m^A^m^ indicates their parental einkorn accessions. The center point, thick line, and thin line below the posterior distribution designate mean, 80%, and 95% credible intervals, respectively. Dashed lines connect center points of each lineage between the synthetic hexaploids and the einkorn accessions. Differences between L1 and L2 (L2 − L1) are shown above the posterior distributions. Asterisks with the differences indicate both the upper 95% credible interval and the lower 95% interval of the difference is above or below zero. Saturation and levels of the grain image were adjusted.

To characterize the grain morphology of the synthetic hexaploids and wild einkorn, we measured five grain-related traits: grain area size, grain length, grain width, grain circularity, and grain perimeter length (S4 and S5 Tables). The grain morphology of L1 in wild einkorn was bigger than that of L2 (Fig 6A). We tested whether differences in grain morphology between the wild einkorn lineages reflected differences in grain morphology between the synthetic hexaploids based on Bayesian statistical models: Model B of GLM and Model C of GLMM. The summary of estimated means and 95% credible intervals of coefficients of each model are shown in S8–S11 Tables. The analysis of Model B showed that the grain length, grain width, grain area size, and grain perimeter length of L2 exhibited smaller values than those of L1 in both wild einkorn and the synthetic hexaploids (Fig 6B). The grain circularity showed opposite values between the wild einkorn and the synthetic hexaploids. The grain circularity of L2 is larger than that of L1 in the wild einkorn, while the grain circularity of L2 is smaller than that of L1. The temperature at the maturity stage influenced grain traits. We incorporated 600°C days as a random variable into the model as Model C. Model C showed better performance than the non-incorporated model (S12 Table). Under the incorporated model, differences in the grain traits became obscure, indicating that the temperature after flowering influenced grain size and morphology (Fig 6C). However, grain length, grain perimeter length, and grain circularity still showed differences in posterior probability distributions between the lineages in both wild einkorn and their synthetic hexaploids.

**Fig 7:**
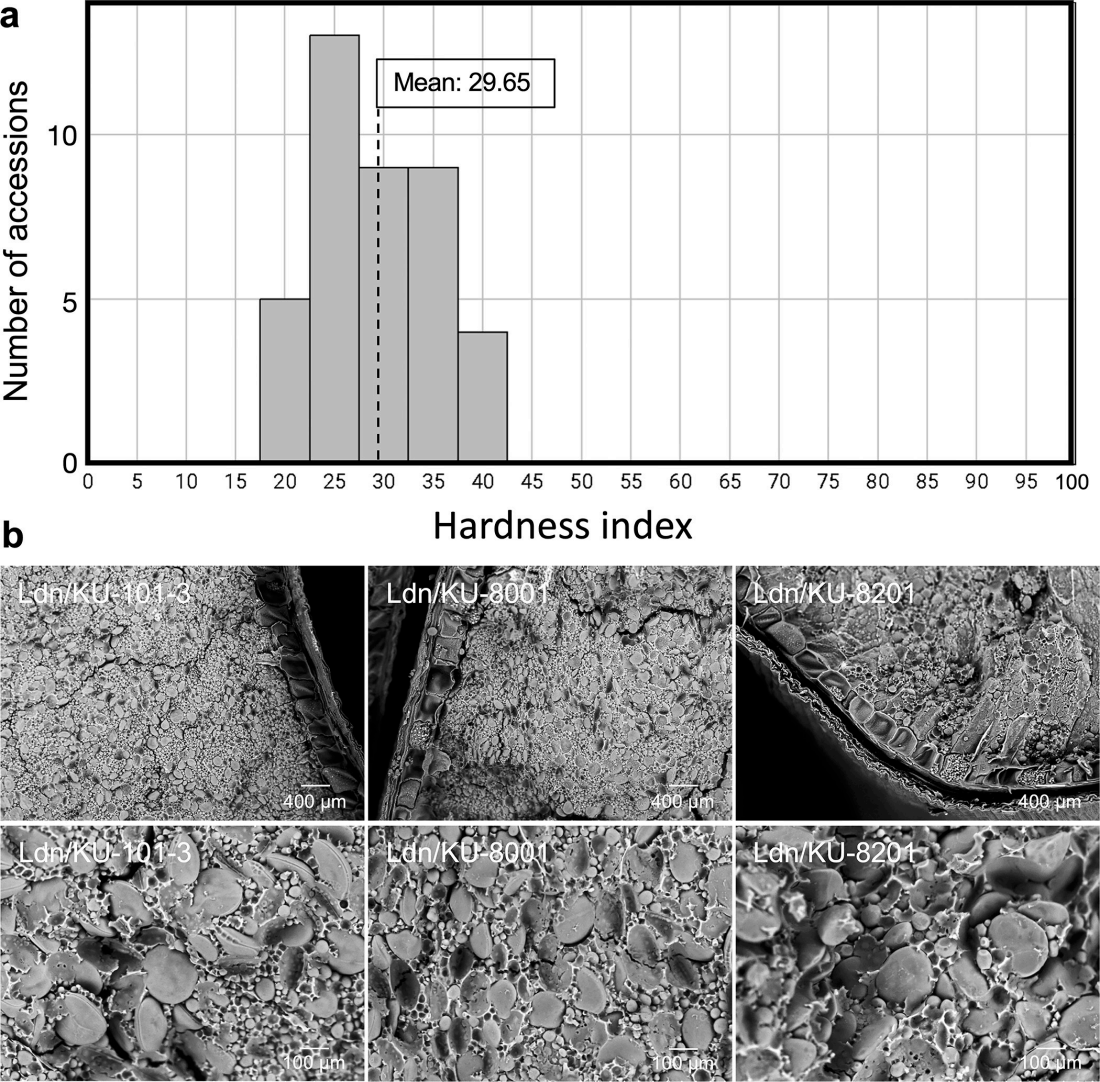
Grain characteristics of the AABBA^m^A^m^ synthetic hexaploids. (a) Frequency distribution of the SKCS hardness values in 40 AABBA^m^A^m^ hexaploid lines (b). Scanning electron microscopy of the transverse sections in Ldn/*Triticum monococcum* ssp. *aegilopoides* KU-101-3, Ldn/*T*. *monococcum* ssp. *aegilopoides* KU-8001, and Ldn/*T*. *monococcum* ssp. *aegilopoides* KU-8201 grains (b). The characteristics of all the AABBA^m^A^m^ hexaploid lines were consistent with those of soft grains of common wheat.

Grain hardness of wheat is an essential parameter that characterizes flour. Hard grain is used for Italian-style pasta, and soft grain is used for other types of noodles, such as ramen and udon noodles. Grain hardness of the 40 AABBA^m^A^m^ synthetic hexaploids, excluding the HDW lines, was measured using a single-kernel characterization system (SKCS), which is algorithmically forced to have a value of 75 for hard wheat and 25 for soft wheat [51]. Grain hardness of the AABBA^m^A^m^ synthetic hexaploids ranged from 18.55 to 42.31 (Fig 7A). Average grain hardness was 29.65 ± 6.00, indicating that grains of the AABBA^m^A^m^ synthetic hexaploids were soft grains.

Scanning electron microscopy was used to observe cross-sections of seeds of three AABBA^m^A^m^ lines (Ldn/ssp. *aegilopoides* KU-101-3, Ldn/ssp. *aegilopoides* KU-8001, and Ldn/ssp. *aegilopoides* KU-8201) (Fig 7B). The starch granule surfaces of all the lines were smooth. We also detected holes formed by starch granules that had fallen out of the endosperm during sample preparation. These observations are consistent with the characteristics of soft grains [32].

### Phenotypic comparisons among AABBA^m^A^m^, AABBAA, and AABBDD synthetic hexaploids

**Fig 8:**
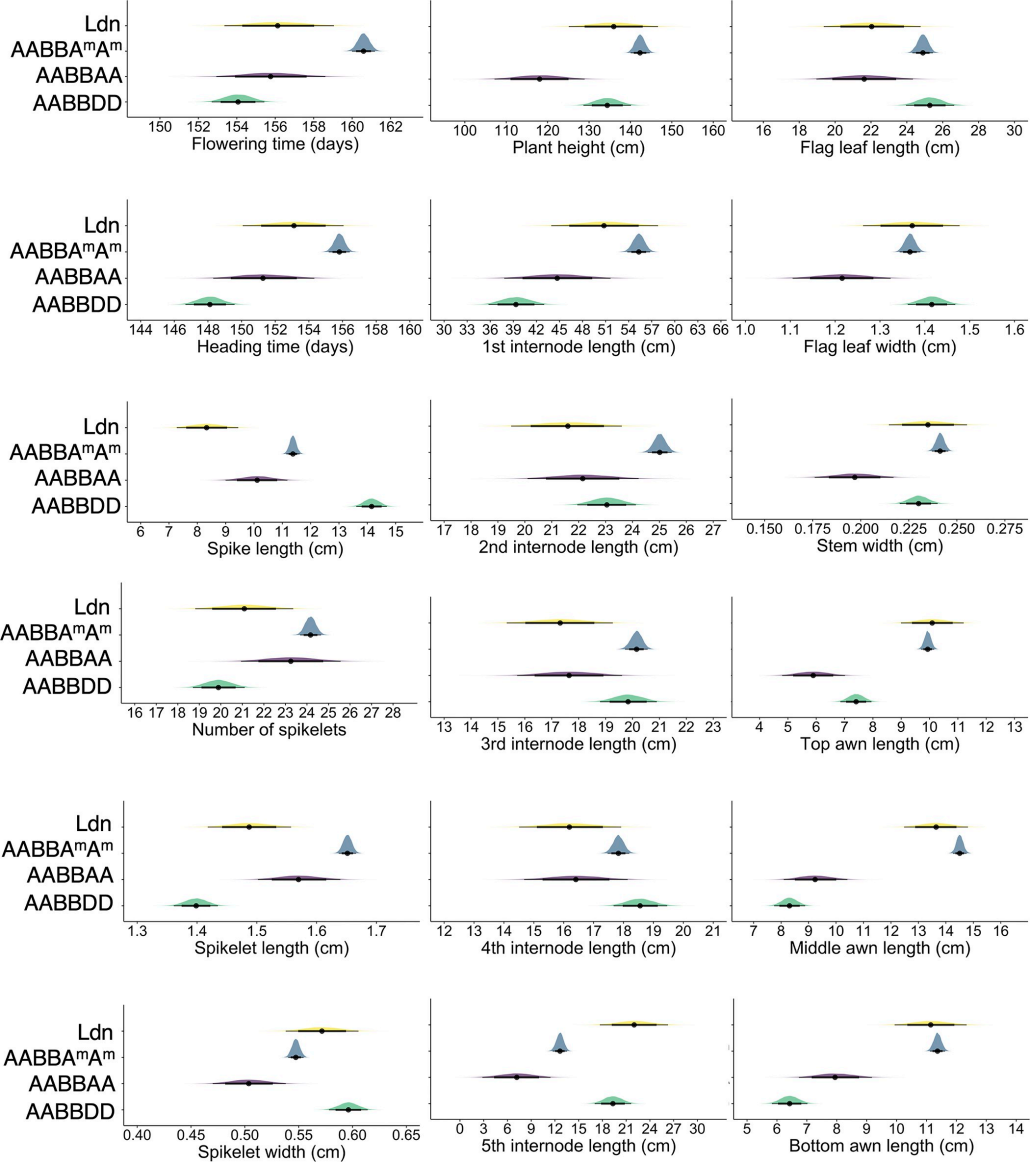
Phenotypic comparisons of the AABB (Ldn), AABBAA, AABBA^m^A^m^, and AABBDD hexaploids. Posterior distributions of mean values for the 18 traits of Ldn and the synthetic hexaploids estimated under Bayesian GLMM. The thick line and thin line below the posterior distribution designate 80% and 95% credible intervals, respectively.

Phenotypic comparisons between AABBDD synthetic wheat and AABBUU synthetic hexaploids indicated that differences in phenotypic traits between these nascent synthetic hexaploids reflect the genomes of their pollen parents [33]. To test whether this observation could extend to the other nascent synthetic hexaploids, we compared the phenotypic traits of AABBDD synthetic wheat with those of the AABBA^m^A^m^ and AABBAA synthetic hexaploids using Model D of Bayesian GLMM (Fig 8). Forty AABBA^m^A^m^ synthetic hexaploids, not including the two HDW synthetic hexaploids, were analyzed by comparison among the synthetic hexaploids. The estimated means and 95% credible intervals of coefficients of Model D are summarized in S13 Table. Heading and flowering dates of the AABBA^m^A^m^ synthetic hexaploids were later than those of the AABBDD synthetic hexaploids. Compared with the AABBDD synthetic wheat, spike length in the AABBA^m^A^m^ synthetic hexaploids was shorter and the number of spikelets was larger, resulting in a high density of spikelets in the AABBA^m^A^m^ synthetic hexaploids. The spikelet shape in the AABBA^m^A^m^ synthetic hexaploids was slenderer than those in the AABBDD synthetic hexaploids. The plant heights of the AABBA^m^A^m^ synthetic hexaploids were larger than those of the AABBDD and AABBAA synthetic hexaploids. In particular, the 1^st^ internode contributed to the plant height in the AABBA^m^A^m^ synthetic hexaploids. However, the 4^th^ and 5^th^ internodes of the AABBDD synthetic hexaploids were longer than those of the AABBA^m^A^m^ synthetic hexaploids. The awn lengths of the AABBA^m^A^m^ synthetic hexaploids were also longer than those of the AABBDD and AABBAA synthetic hexaploids. Flag leaf length and width in the AABBA^m^A^m^ synthetic hexaploids were almost the same as those in the AABBDD synthetic hexaploids. Ldn and the AABBAA synthetic hexaploid showed more broad credible interval than the AABBA^m^A^m^ and AABBDD synthetic hexaploids. Since the number of samples in Ldn and the AABBAA synthetic hexaploid was small due to one accession each, Bayesian GLMM estimated a wide confidence interval centered on the mean of the two seasons.

Since *T*. *monococcum* ssp. *aegilopoides* is known to be genetic resource for providing blue aleurone to common wheat seeds [52], we also tested the color of the grain surface among the synthetic lines based on lab color space: CIE Lab L*, a*, and b* (S7 Fig, S14 Table). Principal component analysis of the lab color space was performed for 39 AABBA^m^A^m^ synthetic hexaploids, Langdon, one AABBAA synthetic hexaploid, and four AABBDD synthetic hexaploids. Some AABBA^m^A^m^ synthetic hexaploids were clearly separated from the AABBDD and AABBAA synthetic hexaploids and Langdon in PC1, which reflects lower CIELab a*, and CIELab b*. This result indicates that these AABBA^m^A^m^ synthetic lines have more grayish grain color than the AABBDD and AABBAA synthetic hexaploids and Langdon.

## Discussion

### Two divergent lineages in *T*. *monococcum* ssp. *aegilopoides* are linked to their habitats

The tested accessions of *T*. *monococcum* ssp. *aegilopoides* were separated into two lineages, L1 and L2, based on polymorphisms for SSR markers that covered all the chromosomes (Fig 1, S2 Table). These separate lineages have also been observed in phylogenetic analysis based on RNA-seq-based polymorphisms [45] and whole-genome polymorphisms in wild einkorn [53]. The habitats of L1 accessions are southern Turkey, northern Iraq, and Iran, which correspond to the Fertile Crescent. L2 accessions are mainly distributed from Greece to Turkey. The genetic differences in wild einkorn reflected their phenotypic traits (Figs 4 and 6). Heading and flowering dates in L2 accessions were later than those in L1 accessions. Flag leaf length and spike length of L2 accessions were longer than those of L1 accessions. The number of spikelets in L2 accessions was larger than that in L1 accessions. These observations suggest that L2 accessions have longer vegetative phases than L1 accessions, allowing for large leaves and a large number of spikelets. On the other hand, the spikelet size of L1 accessions was larger than that of L2 accessions. Grain length, grain width, and grain perimeter length of L1 accessions were also larger than those of L2 accessions. By suppressing the number of spikelets, L1 accessions could invest more nutrients in each of the spikelets and grains, resulting in their larger size.

An amplified fragment length polymorphism (AFLP) analysis for 321 wild einkorn wheat accessions has revealed three distinct einkorn races, “a”, “b”, and “g” [13]. The “a” race is distributed over the Fertile Crescent. The “b” race is observed in a restricted area of southern Turkey. Given that the distribution of the “a” and “b” races overlaps with that of L1, our tested accessions in L1 could belong to the “a” or “b” race. In addition, the habitat of the “g” race is Greece and western Turkey, which overlaps with that of L2. Therefore, this lineage could correspond to the “g” race. Heading date of the “g” race is later than that of the “a” and “b” races. Stem length of the “g” race is longer than that of the “a” and “b” races. The differences in heading date between these einkorn accessions in Kilian et al. (2007) are consistent with the differences between L1 and L2 (Fig 4).

Given that most L1 and L2 accessions were geographically separated, these genetically and morphologically divergent lineages were assumed to have adapted to the local temperature and precipitation of their habitats. Average temperature (°C) per month and average precipitation (mm) per month were estimated in the habitats of each lineage based on WorldClim global climate datasets (S8 Fig). The temperature and precipitation were significantly different between L1 and L2 habitats. The habitat of L1 showed relatively high temperatures and abundant precipitation in winter and rapid drying after May. Due to the high temperatures and sufficient rainfall in winter, the shorter vegetative period of wild einkorn may be sufficient in L1 accessions. The earlier heading and flowering dates in L1 accessions could be essential for reproduction before the start of the dry season. In contrast, L2 accessions are subjected to lower temperatures in winter, which may slow plant growth and necessitate more time for the vegetative period. The habitats of L2 are not as dry as those of L1 in May and June. The delayed flowering of L2 accessions could be a trait naturally selected to adapt to the habitat.

### Transmission of traits from the pollen parents into the nascent synthetic hexaploids

The analysis of phenotypic variations between the AABBA^m^A^m^ synthetic hexaploids and their *T*. *monococcum* ssp. *aegilopoides* pollen parents showed that phenotypic differences between L1 and L2 in the wild einkorn were also present in the synthetic hexaploids (Figs 4 and 6). The synthetic hexaploids derived from L1 accessions had earlier flowering, longer awns, bigger spikelets and grains, and smaller numbers of spikelets than those from L2 accessions. These trait characteristics of the synthetic hexaploids were clearly inherited from their wild einkorn pollen parents. Considering gene dosage effects, the differences between the lineages detected in the wild einkorn are expected to weaken in the synthetic background. However, the differences in the plant height and the internode length unexpectedly became clearer in the hexaploid backgrounds. Our data indicate that the addition of A and B genomes reinforces rather than masks the phenotypic differences in A^m^ genome. In addition, the interseasonal effects on the plant height and the internode length were opposite between the wild einkorn and the synthetic hexaploids (Fig 5). Although the physiological and molecular mechanisms behind these phenomena are unknown, this result indicates that differential responses between synthetic hexaploids and their parental wheat to the environment lead to differences in internode development. Genome-genome interaction in the hexaploid backgrounds may cause this differential response and facilitate lineage differences in internode development.

### Characteristics of the AABBA^m^A^m^ synthetic hexaploids

By comparing the traits among the AABBDD, AABBUU, and AABBA^m^A^m^ synthetic hexaploids sharing the common tetraploid wheat female parent Ldn [33], we found that the phenotypic traits of the AABBA^m^A^m^ synthetic hexaploids are clearly distinct from those of the AABBDD, AABBAA, and AABBUU synthetic hexaploids (Fig 8). The AABBA^m^A^m^ synthetic hexaploids were characterized by higher plant height, more spikelets, slenderer spikelets, longer awns, and relatively late flowering. Given that the same mother parent *T*. *turgidum* cv. Langdon was used to generate the synthetic hexaploids, the phenotypic characteristics of these synthetic hexaploids are considered to reflect those of the diploid species used as their pollen parents. Takumi et al. 2009 [30,54] and Okada et al. 2020 [33] investigated the phenotypic traits of *Ae*. *tauschii* (DD) and *Ae*. *umbellulata* (UU) that were the pollen parents of the AABBDD and AABBUU synthetic hexaploids in the same fields. We compared the phenotypic traits of wild einkorn with *Ae*. *tauschii*, *Ae*. *umbellulata*, and *T*. *urartu* (S15 Table). Wild einkorn had the longest 1^st^ internode, resulting in the tallest plant. Wild einkorn also had the longest awns and bloomed later than the other species. Wild einkorn also had slender spikelets and a large number of spikelets, distinct from the others. Although the phenotypic data for each species were collected in different years, these observations suggest that the phenotypic features of the pollen parents are characteristic of those of the synthetic hexaploids. The large number of spikelets of the AABBA^m^A^m^ hexaploids can be a potentially useful trait to increase yields. The late flowering time of the AABBA^m^A^m^ hexaploids could give more variations of flowering time in common wheat.

The large number of spikelets of the AABBA^m^A^m^ hexaploids can be a potentially useful trait to increase yields. The late flowering time of the AABBA^m^A^m^ hexaploids could give more variations of flowering time in common wheat. Grain hardness of the AABBDD synthetic wheat and the AABBUU synthetic hexaploids was characterized using SKCS and revealed that most of the AABBDD synthetic wheat has soft grains while the AABBUU synthetic hexaploids have hard grains [32,55]. Distribution of grain hardness was compared between these synthetic hexaploids and the AABBA^m^A^m^ hexaploids. Distribution of grain hardness of the AABBA^m^A^m^ hexaploids mostly overlapped with that of the AABBDD synthetic wheat [55]. The distribution of grain hardness of the AABBUU hexaploids was completely separated from that of the AABBA^m^A^m^ hexaploids [33]. The soft grain characteristics of the AABBA^m^A^m^ hexaploids suggest their potential for use in making Asian-style noodles such as udon. Several AABBA^m^A^m^ hexaploids had grayish seeds. This grayish seed color is derived from the wild einkorn. The wild einkorn is known to have blue aleurone containing anthocyanins, of which syntheses were regulated by *Ba2* [56]. The *Ba2* gene of the wild einkorn has been introduced into common wheat [52]. Recently, a transcription factor *TbMYC4A* has been identified as candidate genes of *Ba2* [57]. Since anthocyanin is a functional ingredient for human health as an antioxidant [58], development of wheat with anthocyanin is a target trait of wheat breeding. The AABBA^m^A^m^ hexaploids could be useful genetic resources for developing wheat cultivars containing a higher amount of antioxidant.

The synthetic polyploids often generate aneuploids due to chromosome instability [3,5]. The chromosome instability prevents inheritance of agricultural useful traits to the progenies. Although all the tested AABBA^m^A^m^ hexaploid lines exhibited euploids in our research, it will be essential to evaluate how much chromosome stability AABBA^m^A^m^ hexaploids have compared to synthetic hexaploids with other genome compositions such as AABBDD, AABBUU, and AABBAA through several generations in future.

### Hybrid dwarfness of the AABBA^m^A^m^ synthetic hexaploids

Hybrid dwarfness has been detected in intra- and interspecies hybrids in Triticeae [32,59–61]. Genetic analyses of hybrid dwarfness in wheat indicated that genetic interaction of more than two genes causes hybrid dwarfness [59]. The hybrid dwarfness of the AABBA^m^A^m^ synthetic hexaploids does not exhibit visible necrosis on leaves and grass clump phenotypes. On the other hand, the hybrid dwarfness in the AABBDD synthetic lines exhibits high tiller number and necrosis on leaves under low temperature conditions. Hybrid dwarfness without visible necrosis has not been observed in the AABBDD synthetic hexaploids [61]. The hybrid dwarfness in the AABBUU synthetic hexaploids is characterized by grass clump phenotypes [60,62]. Grass clump dwarfness was also observed in the wheat-rye hybrids. The wheat-rye hybrids stop the elongation of shoot apices and do not produce seeds [60]. Therefore, the symptoms of the hybrid dwarfness in the AABBA^m^A^m^ synthetic hexaploids are distinct from the dwarf phenotypes in the AABBDD and AABBUU synthetic hexaploids and wheat-rye hybrids. According to the studies of intraspecific hybrids in the model plant species of *Arabidopsis thaliana* and rice, autoimmune responses explain the hybrid weakness including hybrid dwarfness and hybrid necrosis [63–66]. For example, the interaction of Toll/interleukin-1 receptor-nucleotide binding-leucine rich repeat genes and a receptor-like kinase gene caused temperature-dependent autoimmunity, resulting in dwarfness [66]. Autoimmunity triggered by the interaction between leucine-rich repeat receptor-like kinase genes and a secreted putative subtilisin-like protease gene causes hybrid dwarfness in rice [65]. Thus, one hypothesis of the hybrid dwarfness of AABBA^m^A^m^ synthetic hexaploids is that autoimmune responses induced by genetic interaction between genes on A or B genome and genes on A^m^ genome cause the hybrid dwarfness. Further molecular genetic studies are required to understand mechanisms behind the hybrid dwarfness in the AABBA^m^A^m^ synthetic hexaploids.

## Conclusions

The analysis of SSR-based polymorphisms in the 43 wild einkorn accessions revealed that wild einkorn genetically and phenotypically diverged into two lineages, L1 and L2. Through interspecific crosses between *T*. *turgidum* cv. Langdon and the wild einkorn accessions, 42 nascent synthetic hexaploids of AABBA^m^A^m^ were generated. The phenotypic differences within the AABBA^m^A^m^ synthetic hexaploids reflected the L1 and L2 divergence among wild einkorn. The introduction of various A^m^ genomes of wild einkorn into the synthetic hexaploids provides increased phenotypic diversity and unique characteristics that can be used for breeding of new and improved wheat cultivars.

## Supporting information

S1 Fig Genotype accumulation curve for the 43 *Triticum monococcum* ssp. *aegilopoides* accessions and the one *T*. *urartu* accession. The horizontal axis represents the number of SSR markers, and the vertical axis shows the number of multilocus genotypes observed in the dataset. The red dashed line represents 100% of the total observed multilocus genotypes.
(PDF)

S2 Fig The full-length gel images in Fig 2. The areas surrounded by the white rectangles correspond to the cropped gel images in Fig 4.
(PDF)

S3 Fig Confirmation of the A^m^-genome chromosomes in the synthetic hexaploids showing hybrid dwarf phenotypes. The presence of the A^m^-genome chromosomes in the synthetic hexaploid lines was confirmed based on amplification of A^m^-chromosome-specific CAPS markers. Their parents (Ldn and wild einkorn accessions) were used as controls. Restriction enzyme and marker names are shown in parentheses following the chromosome names on the left of each gel image. Details of the CAPS markers are described in Table 1. Size differences between the AB and A^m^ genomes were observed. Both amplicons from the AB and A^m^ genomes were detected in the synthetic hexaploid lines. The full-length gel images are shown in S4 Fig.
(PDF)

S4 Fig The full-length gel images in S3 Fig. The areas surrounded by the white rectangles correspond to the cropped gel images in S3 Fig.
(PDF)

S5 Fig Box plots of traits of AABBAmAm accessions showing WT (green) and HDW (orange) phenotypes measured in 2018. Significant differences between two growth phenotypes with Mann-Whitney U-test are marked by asterisks. *p < 0.05, **p < 0.01, ***p < 0.001. NS.: Non-significant.
(PDF)

S6 Fig Box plots of traits of AABBAmAm accessions showing WT (green) and HDW (orange) phenotypes measured in 2019. Significant differences between two growth phenotypes with Mann-Whitney U-test are marked by asterisks. *p < 0.05, **p < 0.01, ***p < 0.001. NS.: Non-significant.
(PDF)

S7 Fig Seed colors of the AABBA^m^A^m^ synthetic hexaploids. (a) Principal component analysis with seed color data (CIELab L*, CIELab A*, and CIELab B*) of 39 synthetic hexaploid lines with the AABBA^m^A^m^ genome (blue circle), one synthetic hexaploid line with the AABBAA genome (red diamond), four synthetic hexaploid lines with the AABBDD genome (green triangle), and Ldn (orange square). (b) Grayish seeds of the AABBA^m^A^m^ synthetic hexaploids and Ldn.
(PDF)

S8 Fig Average temperature and precipitation per month from 1970 to 2000 in the habitats of the L1 and L2 accessions. Significant differences between the habitats of L1 and L2 with Student’s *t*-test are marked by asterisks. *p < 0.05, **p < 0.01, ***p < 0.001. NS: Non-significant.
(PDF)

S1 Table The diploid wheat accessions used in this study. The wild einkorn accessions, except for KU-10725, were used to generate the AABBA^m^A^m^ synthetic hexaploid lines.
(PDF)

S2 Table The SSR markers used in this study. (PDF)

S3 Table Number of alleles, Simpson’s index, expected heterozygosity, and evenness for SSR marker loci used in this study. (PDF)

S4 Table Average trait measurements in *Triticum monococcum* ssp. *aegilopoides* and the AABBA^m^A^m^ synthetic hexaploids. (PDF)

S5 Table Trait data of *Triticum monococcum* ssp. *aegilopoides* and *synthetic hexaploids* in the 2017–2018 season and 2018–2019 season. (XLSX)

S6 Table Summary of posterior means of the fixed coefficients for Bayesian GLMM for the traits of *Triticum monococcum* ssp. *aegilopoides*. (PDF)

S7 Table Summary of posterior means of the fixed coefficients for Bayesian GLMM for the traits of the AABBA^m^A^m^ synthetic hexaploids. (PDF)

S8 Table Summary of posterior means of the fixed coefficients for Bayesian GLM without the parameter of thermal time after anthesis for the grain traits of *Triticum monococcum* ssp. *aegilopoides*. (PDF)

S9 Table Summary of posterior means of the fixed coefficients for Bayesian GLM without the parameter of thermal time after anthesis for the grain traits of the synthetic hexaploids. (PDF)

S10 Table Summary of posterior means of the fixed coefficients for Bayesian GLMM with the parameter of thermal time after anthesis for the grain traits of *Triticum monococcum* ssp. *aegilopoides*. (PDF)

S11 Table Summary of posterior means of the fixed coefficients for Bayesian GLMM with the parameter of thermal time after anthesis for the grain traits of the synthetic hexaploids. (PDF)

S12 Table Results of the model comparisons based on WAIC. (PDF)

S13 Table Summary of posterior means of the fixed coefficients for Bayesian GLMM for the traits between the synthetic hexaploids of AABBA^m^A^m^, AABBAA, and AABBDD. (PDF)

S14 Table Summary of the color of grain surface of Langdon and the synthetic hexaploids with AABBA^m^A^m^ (ABA^m^), AABBAA (ABA), and AABBDD (ABD) genomes. (PDF)

S15 Table Comparison of phenotypic traits among *Triticum monococcum* ssp. *aegilopoides*, *T*. *urartu*, *Aegilops tauschii*, and *Ae*. *umbellulata* that were used as the pollen parents of the synthetic hexaploids. (PDF)
