# Supplementary material for: Phenotypic effects of Am genomes in nascent synthetic hexaploids derived from interspecific crosses between durum and wild einkorn wheat
Source: PLoS One. 2023 Apr 27;18(4):e0284408. doi: 10.1371/journal.pone.0284408 (PMC10138484; doi:10.1371/journal.pone.0284408)
Supplement: S1 Fig — The horizontal axis represents the number of SSR markers, and the vertical axis shows the number of multilocus genotypes observed in the dataset. The red dashed line represents 100% of the total observed multilocus genotypes. (PDF) [file pone.0284408.s001.pdf]

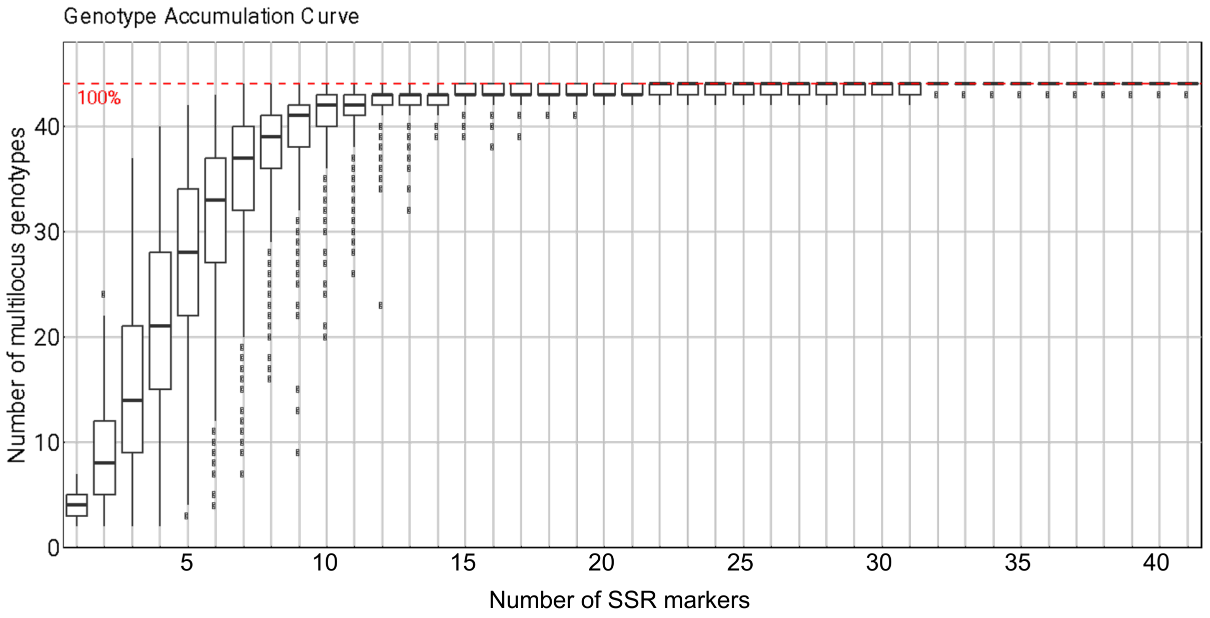

S1 Fig. Genotype accumulation curve for the 43 *Triticum monococcum* ssp. *aegilopoides* accessions and the one *T. urartu* accession

The horizontal axis represents the number of SSR markers, and the vertical axis shows the number of multilocus genotypes observed in the dataset. The red dashed line represents 100% of the total observed multilocus genotypes.
