# Supplementary material for: Phenotypic effects of Am genomes in nascent synthetic hexaploids derived from interspecific crosses between durum and wild einkorn wheat
Source: PLoS One. 2023 Apr 27;18(4):e0284408. doi: 10.1371/journal.pone.0284408 (PMC10138484; doi:10.1371/journal.pone.0284408)
Supplement: S2 Fig — The areas surrounded by the white rectangles correspond to the cropped gel images in Fig 4. (PDF) [file pone.0284408.s002.pdf]

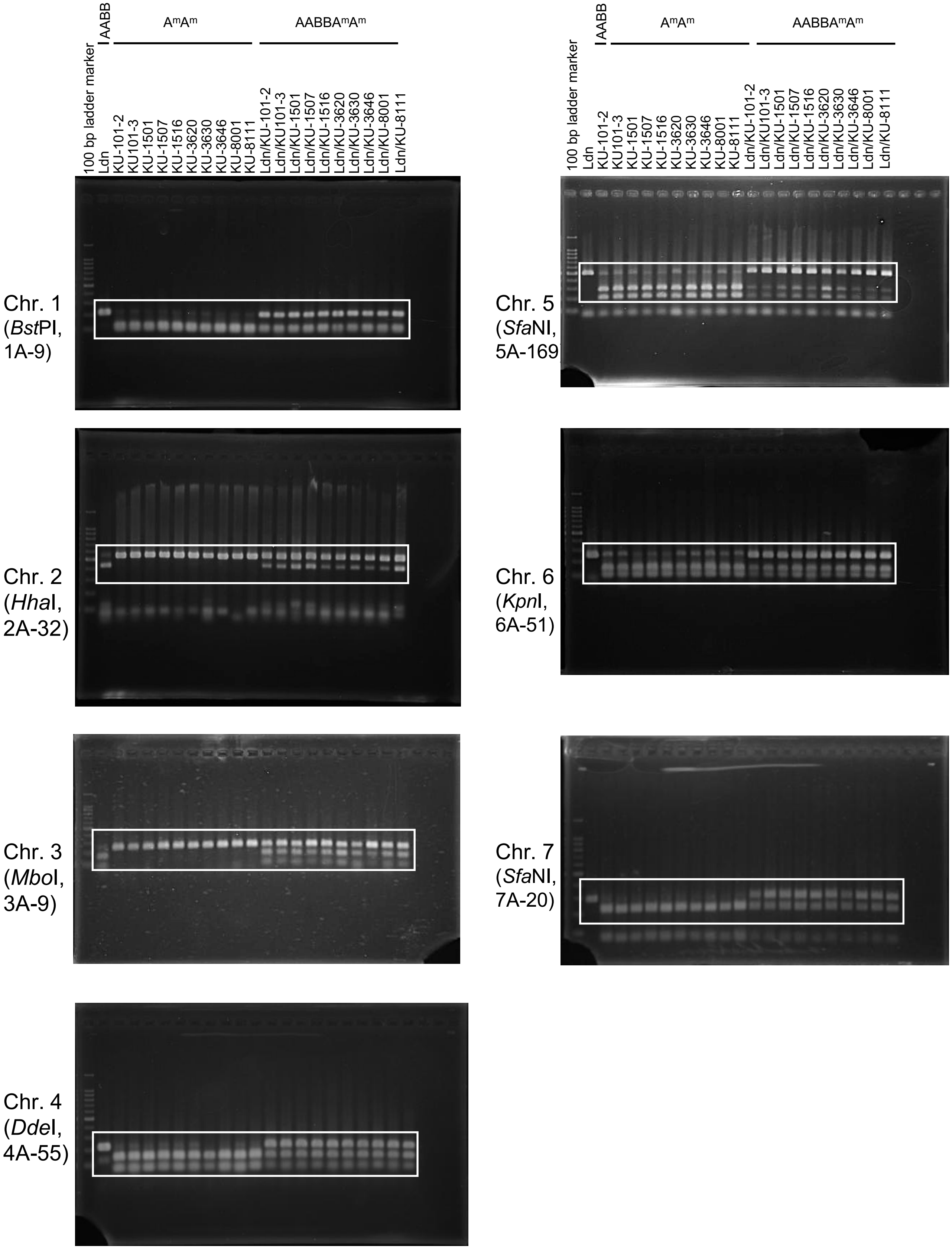

S2 Fig. The full-length gel images in Fig 3

The areas surrounded by the white rectangles correspond to the cropped gel images in Fig 3.

(continued)

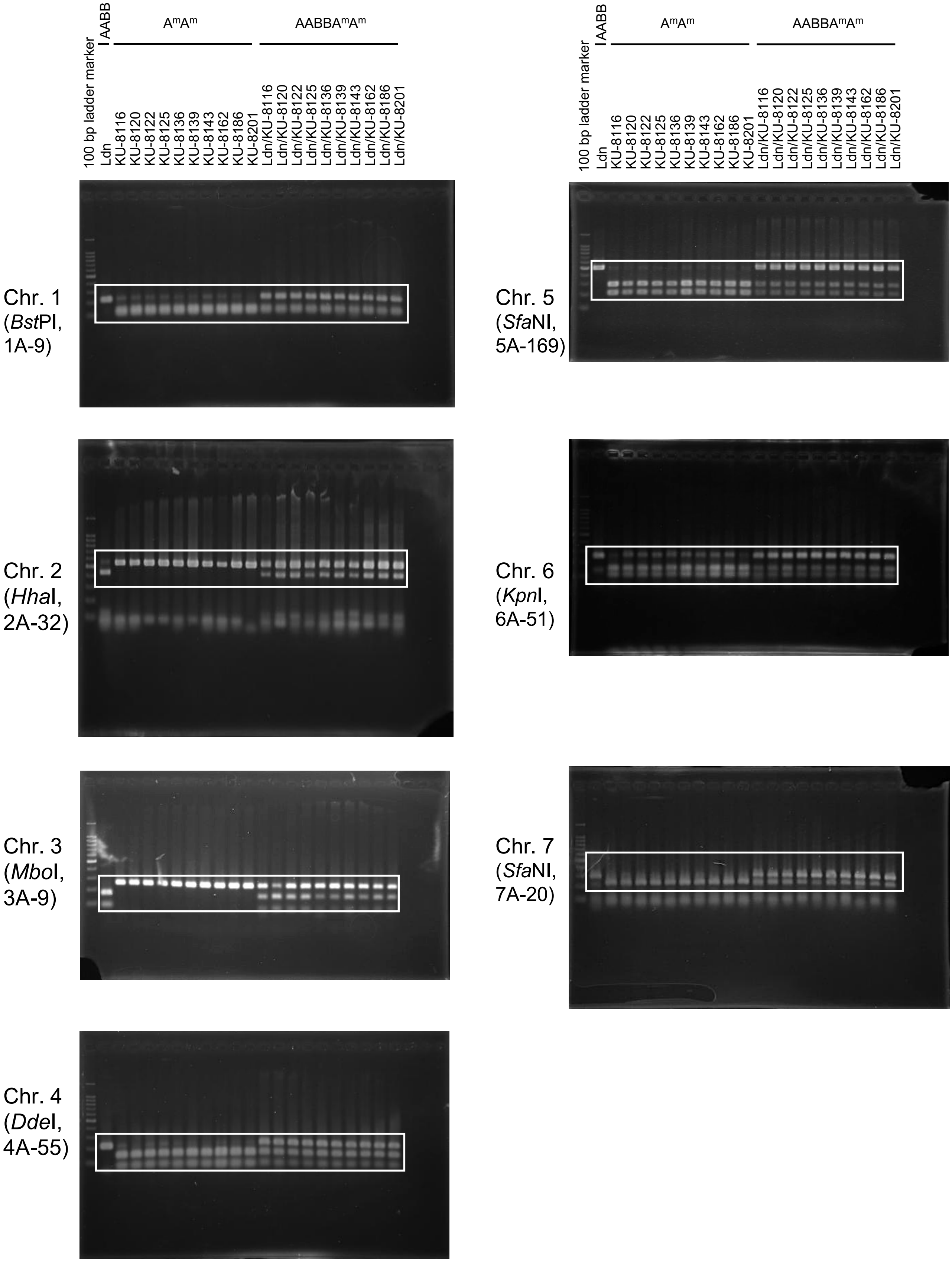

100 bp ladder marker
AABB
Ldn
A^mA^m
KU-8116
KU-8120
KU-8122
KU-8125
KU-8136
KU-8139
KU-8143
KU-8162
KU-8186
KU-8201
$AABBA^mA^m$
Ldn/KU-8116
Ldn/KU-8120
Ldn/KU-8122
Ldn/KU-8125
Ldn/KU-8136
Ldn/KU-8139
Ldn/KU-8143
Ldn/KU-8162
Ldn/KU-8186
Ldn/KU-8201
Chr. 1 (*Bst*PI, 1A-9)
Chr. 2 (*Hha*I, 2A-32)
Chr. 3 (*Mbo*I, 3A-9)
Chr. 4 (*Dde*I, 4A-55)
Chr. 5 (*Sfa*NI, 5A-169)
Chr. 6 (*Kpn*I, 6A-51)
Chr. 7 (*Sfa*NI, 7A-20)

(continued)

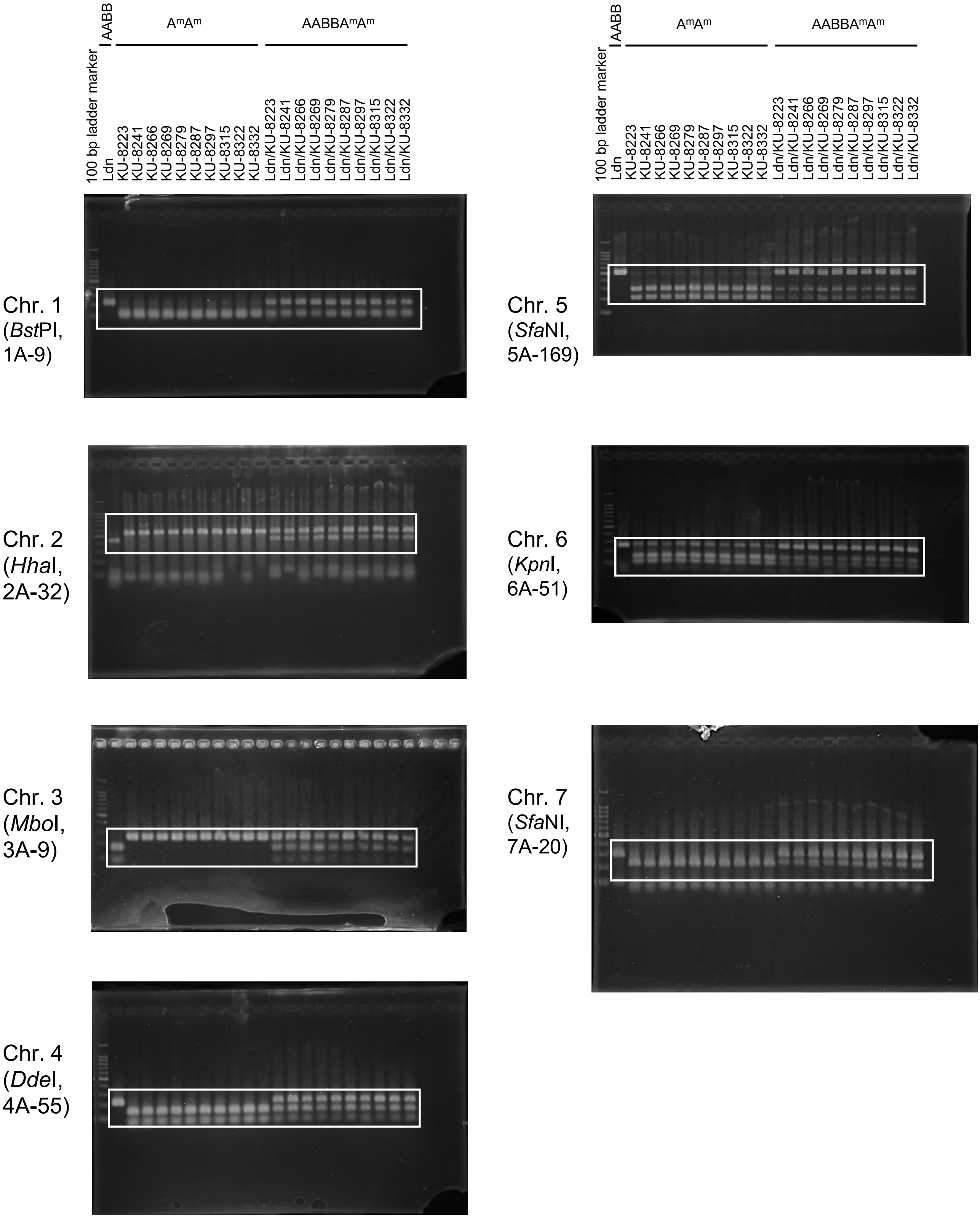

AABB
AmAm
AABBAmAm
100 bp ladder marker
Ldn
KU-8223
KU-8241
KU-8266
KU-8269
KU-8279
KU-8287
KU-8297
KU-8315
KU-8322
KU-8332
Ldn/KU-8223
Ldn/KU-8241
Ldn/KU-8266
Ldn/KU-8269
Ldn/KU-8279
Ldn/KU-8287
Ldn/KU-8297
Ldn/KU-8315
Ldn/KU-8322
Ldn/KU-8332
Chr. 1 (BstPI, 1A-9)
Chr. 2 (HhaI, 2A-32)
Chr. 3 (MboI, 3A-9)
Chr. 4 (DdeI, 4A-55)
Chr. 5 (SfaNI, 5A-169)
Chr. 6 (KpnI, 6A-51)
Chr. 7 (SfaNI, 7A-20)

(continued)

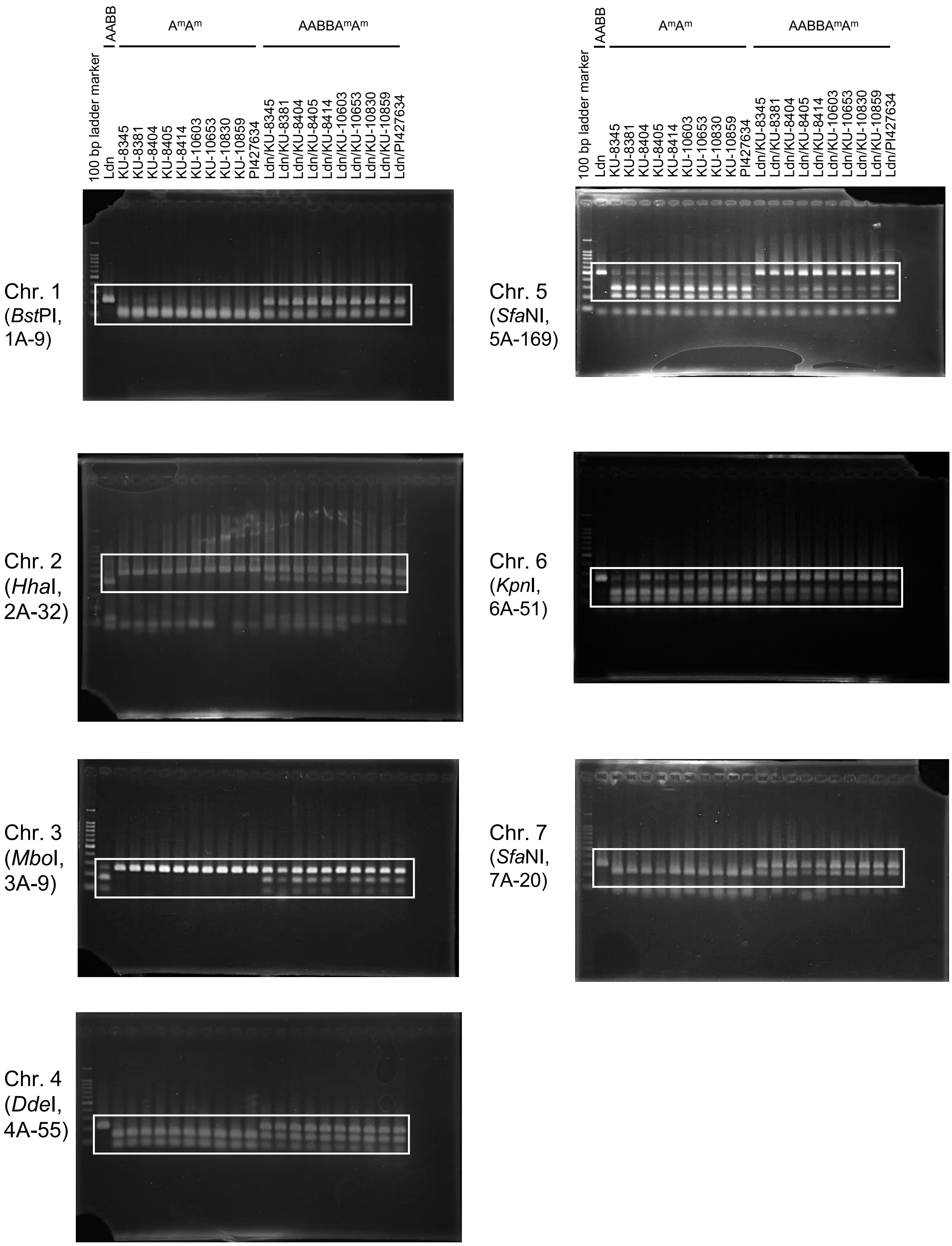

AABB
AmAm
AABBAmAm
100 bp ladder marker
Ldn
KU-8345
KU-8381
KU-8404
KU-8405
KU-8414
KU-10603
KU-10653
KU-10830
KU-10859
PI427634
Ldn/KU-8345
Ldn/KU-8381
Ldn/KU-8404
Ldn/KU-8405
Ldn/KU-8414
Ldn/KU-10603
Ldn/KU-10653
Ldn/KU-10830
Ldn/KU-10859
Ldn/PI427634
Chr. 1 (BstPI, 1A-9)
Chr. 2 (HhaI, 2A-32)
Chr. 3 (MboI, 3A-9)
Chr. 4 (DdeI, 4A-55)
Chr. 5 (SfaNI, 5A-169)
Chr. 6 (KpnI, 6A-51)
Chr. 7 (SfaNI, 7A-20)
