# Supplementary material for: Phenotypic effects of Am genomes in nascent synthetic hexaploids derived from interspecific crosses between durum and wild einkorn wheat
Source: PLoS One. 2023 Apr 27;18(4):e0284408. doi: 10.1371/journal.pone.0284408 (PMC10138484; doi:10.1371/journal.pone.0284408)
Supplement: S3 Fig — The presence of the Am-genome chromosomes in the synthetic hexaploid lines was confirmed based on amplification of Am-chromosome-specific CAPS markers. Their parents (Ldn and wild einkorn accessions) were used as controls. Restriction enzyme and marker names are shown in parentheses following the chromosome names on the left of each gel image. Details of the CAPS markers are described in Table 1. Size differences between the AB and Am genomes were observed. Both amplicons from the AB and Am genomes were detected in the synthetic hexaploid lines. The full-length gel images are shown in S4 Fig. (PDF) [file pone.0284408.s003.pdf]

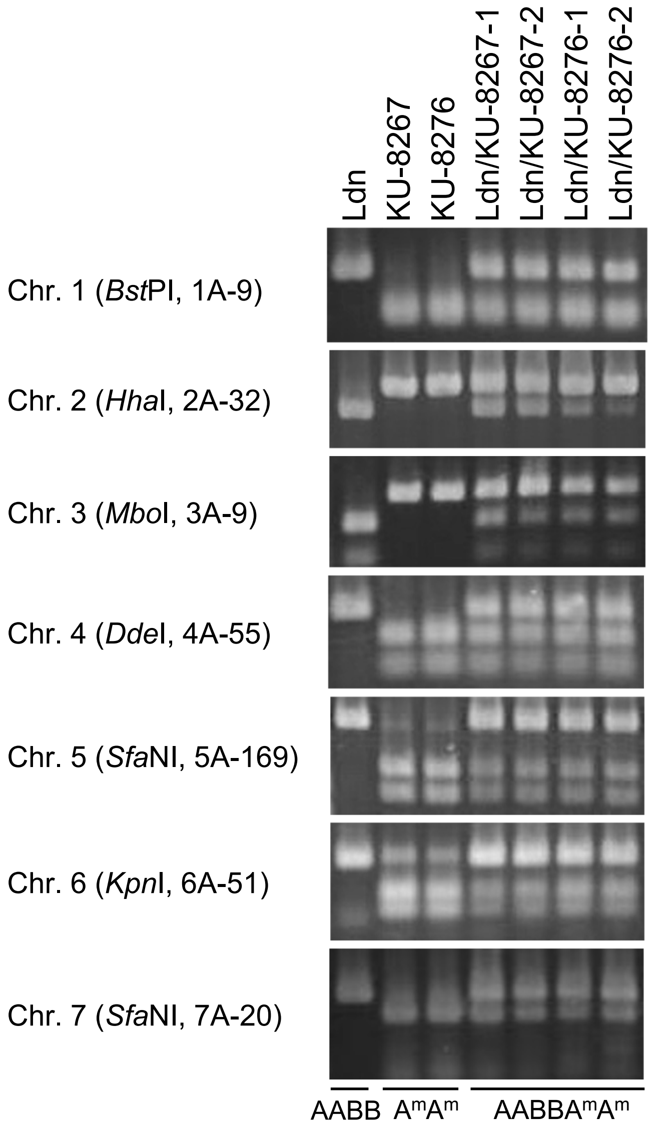

S3 Fig. Wild-type and hybrid dwarf plants of the synthetic allohexaploids

Confirmation of the A^m-genome chromosomes in the synthetic hexaploids showing hybrid dwarf phenotypes. The presence of the A^m-genome chromosomes in the synthetic hexaploid lines was confirmed based on amplification of A^m-chromosome-specific CAPS markers. Their parents (Ldn and wild einkorn accessions) were used as controls. Restriction enzyme and marker names are shown in parentheses following the chromosome names on the left of each gel image. Details of the CAPS markers are described in Table 1. Size differences between the AB and A^m genomes were observed. Both amplicons from the AB and A^m genomes were detected in the synthetic hexaploid lines. The full-length gel images are shown in S4 Fig.
