# Supplementary material for: Phenotypic effects of Am genomes in nascent synthetic hexaploids derived from interspecific crosses between durum and wild einkorn wheat
Source: PLoS One. 2023 Apr 27;18(4):e0284408. doi: 10.1371/journal.pone.0284408 (PMC10138484; doi:10.1371/journal.pone.0284408)
Supplement: S4 Fig — The areas surrounded by the white rectangles correspond to the cropped gel images in S3 Fig. (PDF) [file pone.0284408.s004.pdf]

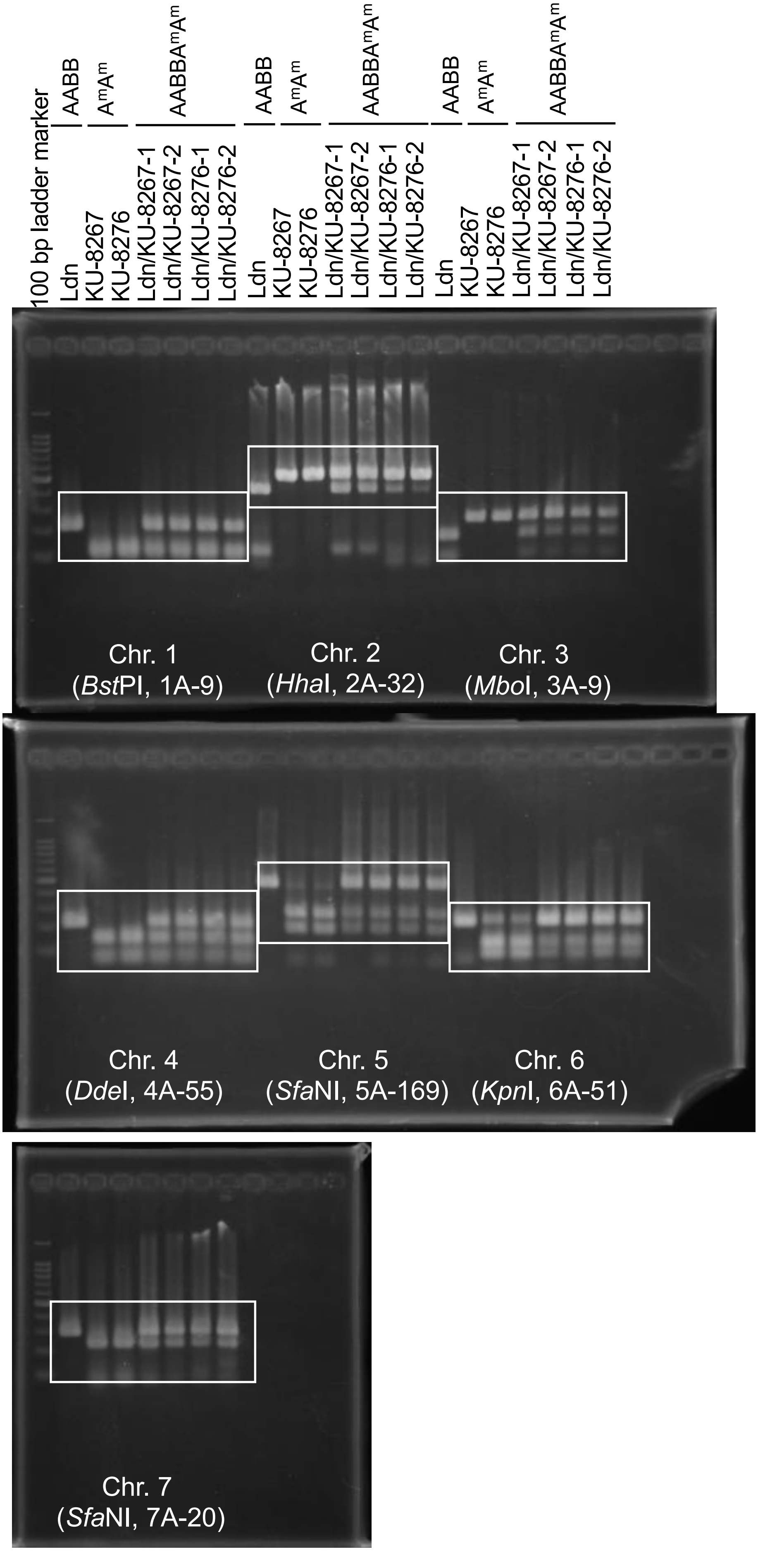

S4 Fig. The full-length gel images in S3 Fig

The areas surrounded by the white rectangles correspond to the cropped gel images in S3 Fig.
