# Supplementary material for: Phenotypic effects of Am genomes in nascent synthetic hexaploids derived from interspecific crosses between durum and wild einkorn wheat
Source: PLoS One. 2023 Apr 27;18(4):e0284408. doi: 10.1371/journal.pone.0284408 (PMC10138484; doi:10.1371/journal.pone.0284408)
Supplement: S6 Fig — Significant differences between two growth phenotypes with Mann-Whitney U-test are marked by asterisks. *p < 0.05, **p < 0.01, ***p < 0.001. NS.: Non-significant. (PDF) [file pone.0284408.s006.pdf]

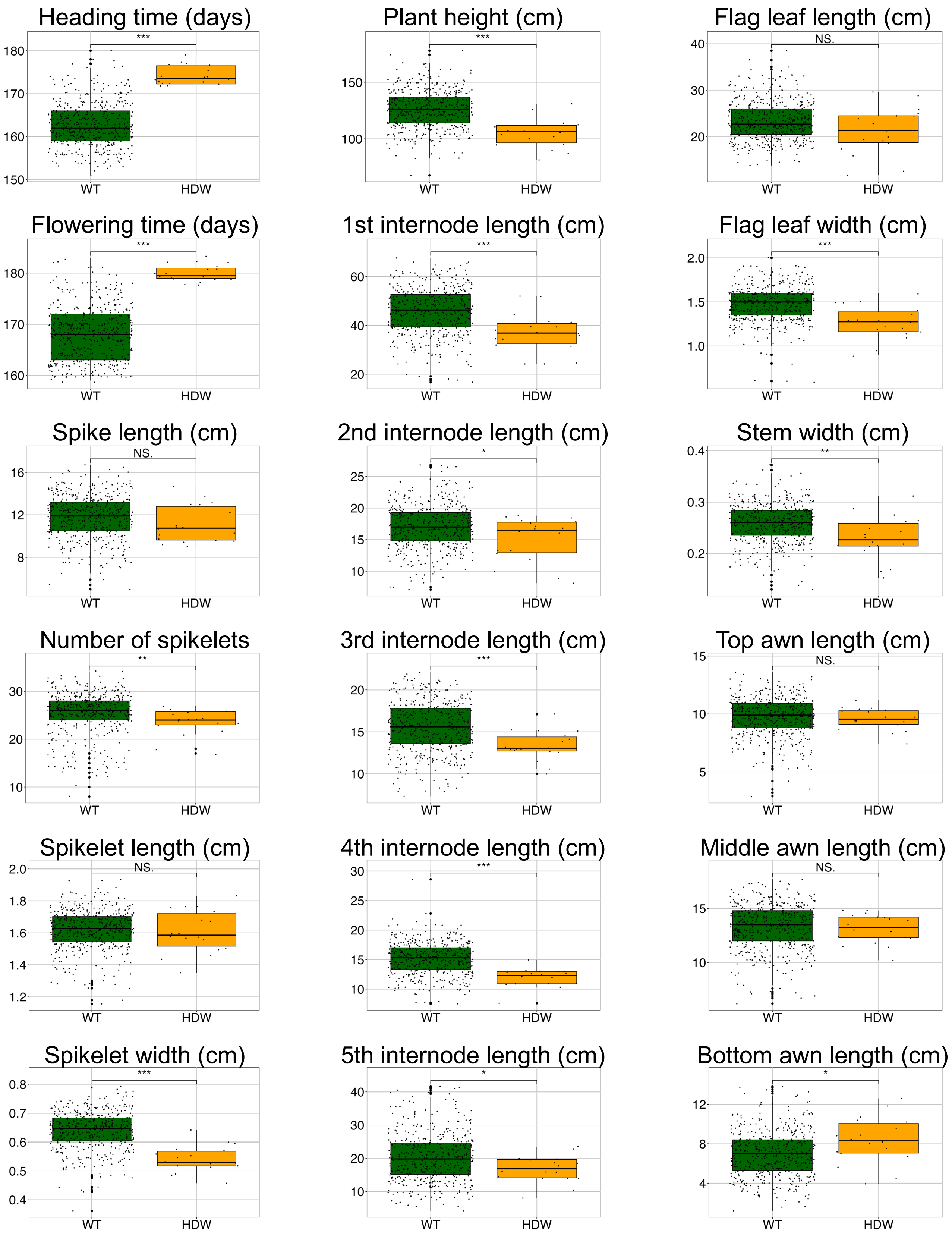

S6 Fig. Box plots of traits of AABBA^{m}A^m accessions showing WT (green) and HDW (orange) phenotypes measured in 2019

Significant differences between two growth phenotypes with Mann-Whitney *U*-test are marked by asterisks. *p < 0.05, **p < 0.01, ***p < 0.001. NS.: non-significant.
