# Supplementary material for: Phenotypic effects of Am genomes in nascent synthetic hexaploids derived from interspecific crosses between durum and wild einkorn wheat
Source: PLoS One. 2023 Apr 27;18(4):e0284408. doi: 10.1371/journal.pone.0284408 (PMC10138484; doi:10.1371/journal.pone.0284408)
Supplement: S7 Fig — (a) Principal component analysis with seed color data (CIELab L*, CIELab A*, and CIELab B*) of 39 synthetic hexaploid lines with the AABBAmAm genome (blue circle), one synthetic hexaploid line with the AABBAA genome (red diamond), four synthetic hexaploid lines with the AABBDD genome (green triangle), and Ldn (orange square). (b) Grayish seeds of the AABBAmAm synthetic hexaploids and Ldn. (PDF) [file pone.0284408.s007.pdf]

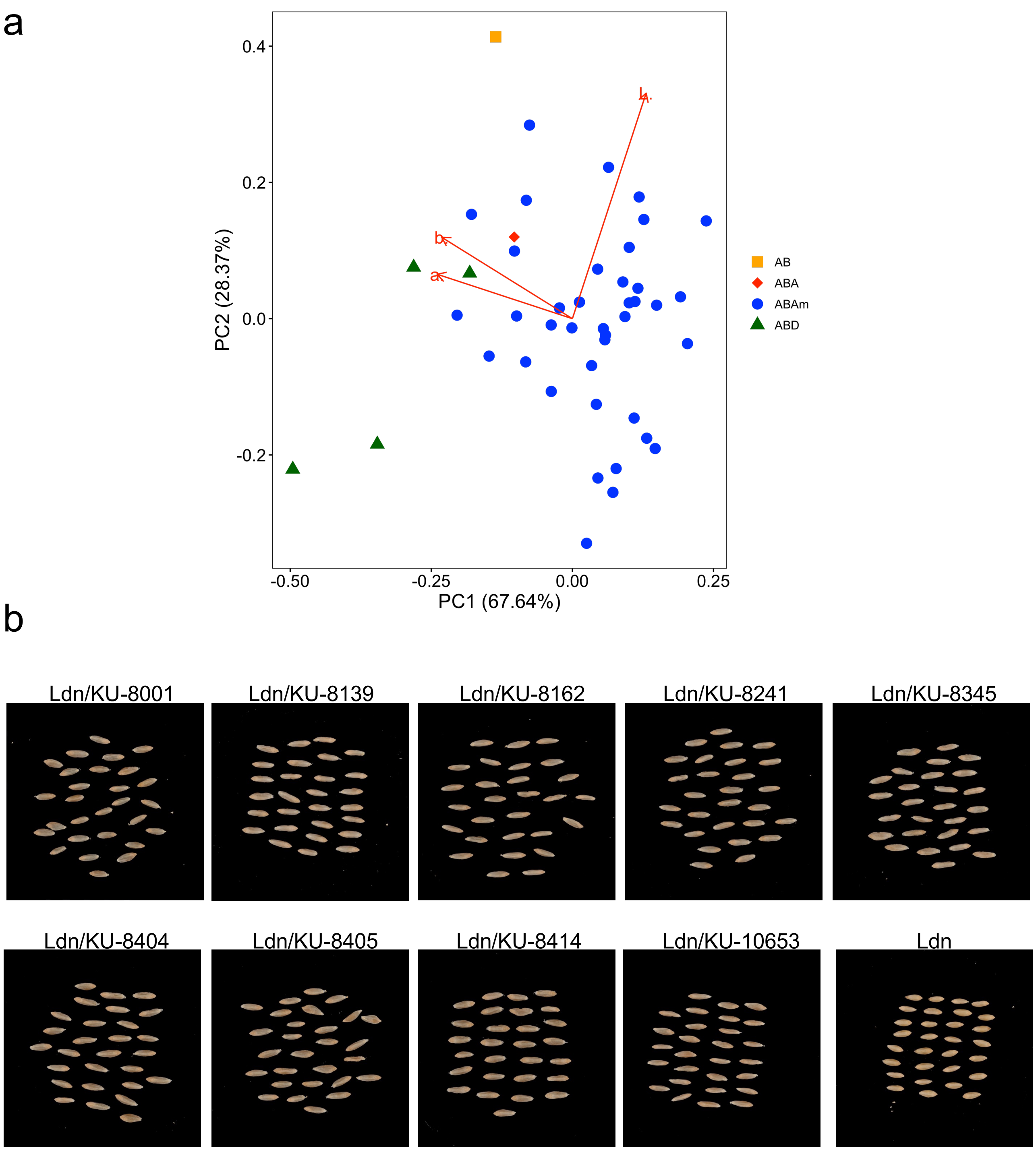

S7 Fig. Seed colors of the AABBA^{m}A^{m} synthetic hexaploids

(a) Principal component analysis with the color of the grain surface (CIELab L*, CIELab A*, and CIELab B*) of 39 synthetic hexaploid lines with the AABBA^{m}A^{m} genome (blue circle), one synthetic hexaploid line with the AABBAA genome (red diamond), four synthetic hexaploid lines with the AABBDD genome (green triangle), and Ldn (orange square). (b) Seed colors of the AABBA^{m}A^{m} synthetic hexaploids and Ldn.
