# Supplementary material for: Phenotypic effects of Am genomes in nascent synthetic hexaploids derived from interspecific crosses between durum and wild einkorn wheat
Source: PLoS One. 2023 Apr 27;18(4):e0284408. doi: 10.1371/journal.pone.0284408 (PMC10138484; doi:10.1371/journal.pone.0284408)
Supplement: S8 Fig — Significant differences between the habitats of L1 and L2 with Student’s t-test are marked by asterisks. *p < 0.05, **p < 0.01, ***p < 0.001. NS: Non-significant. (PDF) [file pone.0284408.s008.pdf]

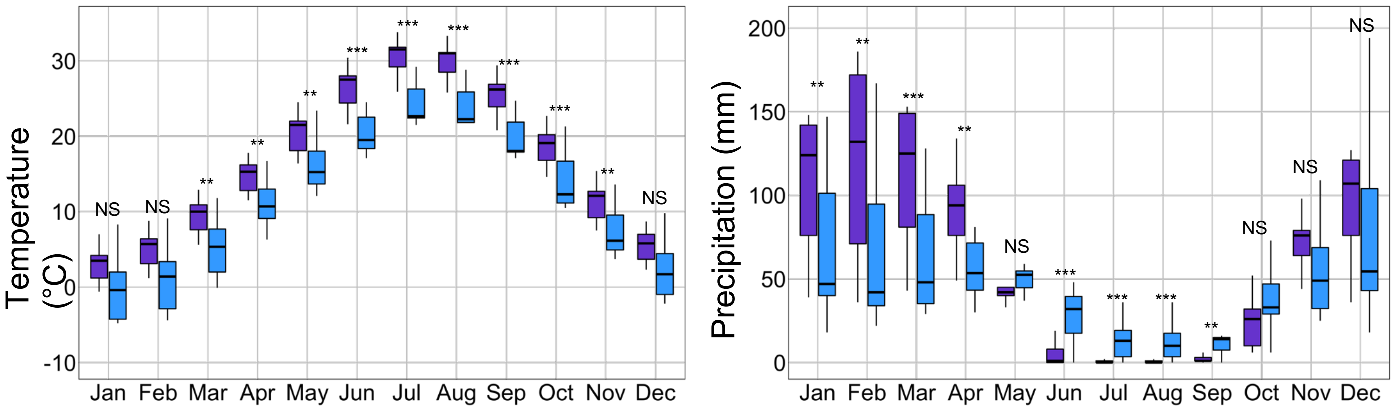

S8 Fig. Average temperature and precipitation per month from 1970 to 2000 in the habitats of the L1 and L2 accessions

Significant differences between the habitats of L1 and L2 with Student's *t*-test are marked by asterisks. $*p < 0.05$, $**p < 0.01$, $***p < 0.001$. NS: non-significant.
