# Supplementary material for: Phenotypic effects of Am genomes in nascent synthetic hexaploids derived from interspecific crosses between durum and wild einkorn wheat
Source: PLoS One. 2023 Apr 27;18(4):e0284408. doi: 10.1371/journal.pone.0284408 (PMC10138484; doi:10.1371/journal.pone.0284408)
Supplement: S1 Table — The wild einkorn accessions, except for KU-10725, were used to generate the AABBAmAm synthetic hexaploid lines. (PDF) [file pone.0284408.s009.pdf]

S1 Table. The diploid wheat accessions used in this study. The wild einkorn accessions, except for KU-10725, were used to generate the AABBA^{m}A^m synthetic hexaploid lines.

Species	Accession number	Longitude	Latitude	Country
Triticum monococcum ssp. *aegilopoides*	KU-101-2	33.60021263	44.50019923	Ukraine
	KU-101-3	51.42305149	35.69611582	Iran
	KU-1501	44.51620226	40.18420457	Armenia
	KU-1507	44.72284657	40.11954847	Armenia
	KU-1516	44.72284657	40.11954847	Armenia
	KU-3620	32.85603022	39.94477358	Turkey
	KU-3630	19.84678983	39.79691512	Greece
	KU-3646	38.79299849	37.15120472	Turkey
	KU-8001	45.45028086	35.5551134	Iraq
	KU-8111	44.64717134	36.08438954	Iraq
	KU-8116	44.64724517	36.08439408	Iraq
	KU-8120	44.6473035	36.08439867	Iraq
	KU-8122	44.64739401	36.08439501	Iraq
	KU-8125	44.35082629	36.40061086	Iraq
	KU-8136	44.35028295	36.40062703	Iraq
	KU-8139	44.35036366	36.40062703	Iraq
	KU-8143	44.35048243	36.40062703	Iraq
	KU-8162	44.54039534	36.65050752	Iraq
	KU-8186	44.35064338	36.40062703	Iraq
	KU-8201	44.35079709	36.40062703	Iraq
	KU-8223	41.73398655	36.61756221	Syria
	KU-8241	43.48892794	37.09297549	Iraq
	KU-8266	43.48866064	37.09324742	Iraq
	KU-8267*	42.68332093	37.14997658	Iraq
	KU-8269	42.68342374	37.15002361	Iraq
	KU-8276*	40.74409963	37.31315101	Turkey
	KU-8279	40.22573554	37.91612383	Turkey
	KU-8287	40.22605428	37.91612066	Turkey
	KU-8297	39.22299452	38.6756018	Turkey
	KU-8315	38.31520009	38.34750116	Turkey
	KU-8322	39.22369996	38.67560746	Turkey
	KU-8332	40.67609577	39.04110576	Turkey
	KU-8345	40.67637342	39.04130576	Turkey
	KU-8381	43.37325669	38.50122343	Turkey
	KU-8404	46.99492983	35.31871231	Iran
	KU-8405	46.99502177	35.31871004	Iran
	KU-8414	46.24696566	34.28032449	Iran
	KU-10603	32.32070473	40.07931297	Turkey
	KU-10653	41.00138477	38.14222317	Turkey
	KU-10725	32.85409987	39.92077215	Turkey
	KU-10830	38.30946155	38.3551938	Turkey
	KU-10859	36.93332881	37.58333508	Turkey
	PI427634	39.49985	37.717213	Turkey
Triticum urartu	KU-199-16	36.16941521	34.02132731	Lebanon

Asterisks indicate the HDW-showing ABA^m hexaploid lines.
