# Supplementary material for: Phenotypic effects of Am genomes in nascent synthetic hexaploids derived from interspecific crosses between durum and wild einkorn wheat
Source: PLoS One. 2023 Apr 27;18(4):e0284408. doi: 10.1371/journal.pone.0284408 (PMC10138484; doi:10.1371/journal.pone.0284408)
Supplement: S2 Table — (PDF) [file pone.0284408.s010.pdf]

S2 Table. The SSR markers used in this study.

Marker name	Chromosome arm	Forward and reverse primer sequences (5' to 3')	Marker type	Annealing temp. (ºC)
gwm33	1AS	GGAGTCACACTTGTTTGTGCA<br>CACTGCACACCTAACTACCTGC	SSR	58
barc83	1AS	AAGCAAGGAACGAGCAAGAGCAGTAG<br>TGGATTTACGACGACGATGAAGATGA	SSR	58
gwm135	1AL	TGTCAACATCGTTTTGAAAAGG<br>ACACTGTCAACCTGGCAATG	SSR	58
wmc278	1AL	AAACGATAGTAAAATTACCTCGGAT<br>TCAAAAAATAGCAACTTGAAGACAT	SSR	58
cfa2219	1AL	TCTGCCGAGTCACTTCATTG<br>GACAAGGCCAGTCCAAAAGA	SSR	56
gwm512	2AS	AGCCACCATCAGCAAAAATT<br>GAACATGAGCAGTTTGGCAC	SSR	58
gwm296	2AS	AATTCAACCTACCAATCTCTG<br>GCCTAATAAACTGAAAACGAG	SSR	58
wmc602	2AS	TACTCCGCTTTGATATCCGTCC<br>GTTTGTTGTTGCCATCACATTC	SSR	58
wmc522	2AS	AAAAATCTCACGAGTCGGGC<br>CCCGAGCAGGAGCTACAAAT	SSR	58
wmc474	2AS	ATGCTATTAAACTAGCATGTGTCG<br>AGTGGAAACATCATTCCTGGTA	SSR	58
gwm558	2AL	GGGATTGCATATGAGACAACG<br>TGCCATGGTTGTAGTAGCCA	SSR	58
barc5	2AL	GCGCCTGGACCGGTTTTCTATTTT<br>GCGTTGGGAATTCCTGAACATTTT	SSR	58
gwm356	2AL	AGCGTTCTTGGGAATTAGAGA<br>CCAATCAGCCTGCAACAAC	SSR	58
wmc532	3AS	GATACATCAAGATCGTGCCAAA<br>GGGAGAAATCATTAACGAAGGG	SSR	58
wmc664	3AS	GGGCCAACAAATCCAAT<br>TCTACTTCCTTCATCCACTCC	SSR	58
hbg345	3AL	CTAGACGTTTGCATACAAATTTCCG<br>CAGGGCTCATCACATTTGCAATTTG	SSR	58
cfa2193	3AL	ACATGTGATGTGCGGTCATT<br>TCCTCAGAACCCCATTCTTG	SSR	58
wmc169	3AL	TACCCGAATCTGGAAAATCAAT<br>TGGAAGCTTGCTAACTTTGGAG	SSR	58
gwm165	4AS	TGCAGTGGTCAGATGTTTCC<br>CTTTTCTTTCAGATTGCGCC	SSR	58
barc206	4AS	GCTTTGCCAGGTGAGCACTCT<br>TGGCCGGGTATTTGAGTTGGAGTTT	SSR	58
gwm44	4AL	GTTGAGCTTTTCAGTTCGGC<br>ACTGGCATCCACTGAGCTG	SSR	58
cfa2256	4AL	GGTAATATTCAGGTTACCGCACA<br>GGTAAAGTTATAAATTGTTGTGGGC	SSR	58
wmc468	4AL	AGCTGGGTTAATAACAGAGGAT<br>CACATAACTGTCCACTCCTTTC	SSR	58
barc70	4AL	GCGAAAAACGATGCGACTCAAAG<br>GCGCCATATAATTCAGACCCACAAAA	SSR	56

Marker positions were referenced to Somers et al. (2014) and Torada et al. (2006).

Table S2 (Continued)

Marker name	Chromosome arm	Forward and reverse primer sequences (5' to 3')	Marker type	Annealing temp. (ºC)
gwm443	5AS	GGGTCTTCATCCGGAACTCT<br>CCATGATTTATAAATTCCACC	SSR	58
gwm293	5AS	TACTGGTTCACATTGGTGCG<br>TCGCCATCACTCGTTCAAG	SSR	58
gwm186	5AS	GCAGAGCCTGGTTCAAAAAG<br>CGCCTCTAGCGAGAGCTATG	SSR	58
gwm156	5AL	CCAACCGTGCTATTAGTCATTC<br>CAATGCAGGCCCTCCTAAC	SSR	58
gwm617	5AL	GATCTTGGCGCTGAGAGAGA<br>CTCCGATGGATTACTCGCAC	SSR	58
cfa2155	5AL	TTTGTTACAACCCAGGGGG<br>TTGTGTGGCGAAAGAAACAG	SSR	58
gwm291	5AL	CATCCCTACGCCACTCTGC<br>AATGGTATCTATTCCGACCCG	SSR	56
barc146	6A	AAGGCGATGCTGCAGCTAAT<br>GGCAATATGGAAACTGGAGAGAAAT	SSR	58
wmc553	6AL	CGGAGCATGCAGCTAGTAA<br>CGCCTGCAGAATTCAACAC	SSR	58
wmc417	6AL	GTTCTTTTAGTTGCGACTGAGG<br>CGATGTATGCCGTATGAATGTT	SSR	58
gwm427	6AL	AAACTTAGAACTGTAATTTCAGA<br>AGTGTGTTCATTTGACAGTT	SSR	58
cfd13	7AS	CCACTAACCAAGCTGCCATT<br>TTTTTGGCATTGATCTGCTG	SSR	58
cfd242	7AS	CCAGTTTGCAGCAGTCACAT<br>CAGACCTTAACGGGGTTGAA	SSR	58
barc154	7AS	GTAATTCCGGTTCCACTTGACATT<br>GGATGGGCAGCTTCAAGGTATGTT	SSR	58
cfa2028	7AS	TGGGTATGAAAGGCTGAAGG<br>ATCGCGACTATTCAACGCTT	SSR	58
barc174	7AS	TGGCATTTTTCTAGCACCAATACAT<br>GCGAACTGGACCAGCCTTCTATCTGTTC	SSR	58
wmc607	7AS	ATATATGCCCATGAAGCTCAAG<br>GATCGAGCTAAAGCTGATACCA	SSR	58
gwm332	7AS	AGCCAGCAAGTCACCAAAAC<br>AGTGCTGGAAAGAGTAGTGAAGC	SSR	58
