# Supplementary material for: Phenotypic effects of Am genomes in nascent synthetic hexaploids derived from interspecific crosses between durum and wild einkorn wheat
Source: PLoS One. 2023 Apr 27;18(4):e0284408. doi: 10.1371/journal.pone.0284408 (PMC10138484; doi:10.1371/journal.pone.0284408)
Supplement: S3 Table — (PDF) [file pone.0284408.s011.pdf]

S3 Table. Number of alleles, Simpson's index, expected heterozygosity, and evenness for SSR marker loci used in this study.

SSR markers	Number of alleles	Simpson's index	Expected heterozygosity	Evenness
gwm33	4	0.6333	0.6480	0.8469
barc83	2	0.0444	0.0455	0.4057
gwm135	4	0.6901	0.7061	0.8614
wmc278	5	0.7180	0.7347	0.8883
cfa2219	4	0.5837	0.5973	0.7745
gwm512	2	0.4959	0.5074	0.9918
gwm296	2	0.0444	0.0455	0.4057
wmc602	5	0.6188	0.6332	0.7704
wmc522	2	0.0444	0.0455	0.4057
wmc474	2	0.0444	0.0455	0.4057
gwm558	4	0.6126	0.6268	0.7837
barc5	6	0.7107	0.7273	0.7045
gwm356	3	0.1291	0.1321	0.4367
wmc532	3	0.6043	0.6184	0.9001
wmc664	3	0.3048	0.3118	0.6083
hbg345	3	0.5196	0.5317	0.7649
cfa2193	7	0.8110	0.8298	0.8456
wmc169	3	0.4907	0.5021	0.8568
gwm165	3	0.6353	0.6501	0.9381
barc206	2	0.0444	0.0455	0.4057
gwm44	2	0.4494	0.4598	0.9072
cfa2256	3	0.2407	0.2463	0.5431
wmc468	4	0.6684	0.6839	0.9169
barc70	5	0.4649	0.4757	0.5835
gwm443	6	0.7107	0.7273	0.7073
gwm293	2	0.0444	0.0455	0.4057
gwm186	4	0.6498	0.6649	0.7848
gwm156	7	0.7986	0.8171	0.8841
gwm617	6	0.6674	0.6829	0.6714
cfa2155	5	0.6374	0.6522	0.7104
gwm291	2	0.0444	0.0455	0.4057
barc146	5	0.4432	0.4535	0.5515
wmc553	4	0.5475	0.5603	0.7277
wmc417	3	0.3048	0.3118	0.6083
gwm427	6	0.6715	0.6871	0.7800
cfd13	4	0.7107	0.7273	0.9275
cfd242	3	0.4638	0.4746	0.8123
barc154	3	0.6570	0.6723	0.9786
cfd2028	5	0.6622	0.6776	0.7127
barc174	5	0.7800	0.7981	0.9394
wmc607	2	0.0444	0.0455	0.4057
gwm332	2	0.0444	0.0455	0.4057

S3 Table. (Continued)

SSR markers	Number of alleles	Simpson's index	Expected heterozygosity	Evenness
Mean of chr. 1	3.800	0.5339	0.5463	0.7554
Mean of chr. 2	3.250	0.3376	0.3454	0.6130
Mean of chr. 3	3.800	0.5461	0.5588	0.7952
Mean of chr. 4	3.167	0.4172	0.4269	0.7158
Mean of chr. 5	4.571	0.5075	0.5193	0.6528
Mean of chr. 6	4.500	0.4917	0.5032	0.6669
Mean of chr. 7	3.429	0.4804	0.4915	0.7403
Mean of all chr.	3.738	0.4639	0.4747	0.6993
