# Supplementary material for: Phenotypic effects of Am genomes in nascent synthetic hexaploids derived from interspecific crosses between durum and wild einkorn wheat
Source: PLoS One. 2023 Apr 27;18(4):e0284408. doi: 10.1371/journal.pone.0284408 (PMC10138484; doi:10.1371/journal.pone.0284408)
Supplement: S4 Table — (PDF) [file pone.0284408.s012.pdf]

**S4 Table.** Average trait measurements in *Triticum monococcum* ssp. *aegilopoides* and the AABBA$^{m}$A$^{m}$ synthetic hexaploids.

| Traits | *T. monococcum* ssp. *aegilopoides* | | Synthetic hexaploids | |
|---|---|---|---|---|
| | 2017 – 2018 | 2018 – 2019 | 2017 – 2018 | 2018 – 2019 |
| Heading time (days) | 170 ± 9.99 | 179 ± 9.49 | 156 ± 2.99 | 162 ± 4.60 |
| Flowering time (days) | 176 ± 9 | 186 ± 8.59 | 160 ± 2.12 | 168 ± 4.87 |
| Spike length (cm) | 9.69 ± 1.411 | 10.99 ± 1.82 | 11.58 ± 1.29 | 12.29 ± 1.74 |
| Number of spikelets | 25.1 ± 4.44 | 31.9 ± 5.76 | 24.5 ± 2.73 | 26.9 ± 3.09 |
| Spikelet length (cm) | 1.18 ± 0.01 | 1.23 ± 0.09 | 1.67 ± 0.09 | 1.63 ± 0.10 |
| Spikelet width (cm) | 0.263 ± 0.03 | 0.293 ± 0.03 | 0.544 ± 0.04 | 0.653 ± 0.05 |
| Plant height (cm) | 81.3 ± 11.7 | 100 ± 17.10 | 147.1 ± 16.00 | 128 ± 15.84 |
| 1st Internode length (cm) | 31 ± 7.48 | 40 ± 8.38 | 57.5 ± 11.65 | 47.4 ± 8.65 |
| 2nd Internode length (cm) | 16.4 ± 2.96 | 19 ± 3.75 | 25.8 ± 3.05 | 17.3 ± 3.07 |
| 3rd Internode length (cm) | 11.18 ± 1.8 | 13.32 ± 2.48 | 20.8 ± 2.81 | 15.7 ± 2.96 |
| 4th Internode length (cm) | 8.18 ± 1.58 | 10.2 ± 2.65 | 18.28 ± 2.55 | 15.1 ± 2.78 |
| 5th Internode length (cm) | 5.02 ± 2.88 | 6.61 ± 4.00 | 13.08 ± 7.14 | 20.3 ± 7.03 |
| Flag leaf length (cm) | 6.75 ± 2.025 | 12.7 ± 4.09 | 25 ± 4.62 | 23.4 ± 4.14 |
| Flag leaf width (cm) | 0.513 ± 0.1069 | 0.761 ± 0.14 | 1.366 ± 0.16 | 1.48 ± 0.17 |
| Top awn length (cm) | 6.42 ± 2.985 | 8.61 ± 1.69 | 10.02 ± 2.06 | 9.7 ± 1.60 |
| Middle awn length (cm) | 10.37 ± 2.7 | 9.72 ± 2.33 | 14.59 ± 1.61 | 13.26 ± 1.99 |
| Bottom awn length (cm) | 9.06 ± 2.237 | 4.02 ± 2.06 | 11.74 ± 1.46 | 6.93 ± 2.16 |
| Stem width (cm) | 0.119 ± 0.021 | 0.153 ± 0.02 | 0.246 ± 0.03 | 0.265 ± 0.03 |
| Grain length (mm) | 6.65 ± 0.75 | | 8.84 ± 0.78 | |
| Grain width (mm) | 1.22 ± 0.26 | | 2.13 ± 0.36 | |
| Grain perimeter length (mm) | 15.2 ± 1.75 | | 20.3 ± 1.84 | |
| Grain area size (mm$^2$) | 6.09 ± 1.65 | | 13.9 ± 2.87 | |
| Grain circularity | 0.327 ± 0.05 | | 0.42 ± 0.05 | |
