# Supplementary material for: Phenotypic effects of Am genomes in nascent synthetic hexaploids derived from interspecific crosses between durum and wild einkorn wheat
Source: PLoS One. 2023 Apr 27;18(4):e0284408. doi: 10.1371/journal.pone.0284408 (PMC10138484; doi:10.1371/journal.pone.0284408)
Supplement: S6 Table — (PDF) [file pone.0284408.s014.pdf]

S6Table. Summary of posterior means of the fixed coefficients for Bayesian GLMM for the traits of *Triticum monococcum* ssp. *aegilopoides*.

Traits	Effects	Estimate	Est.Error	l-95% CI	u-95% CI	Rhat	Bulk ESS	Tail ESS
Heading time (days)	sd (ID)	8.634	0.529	7.657	9.748	1.00	1757.1	3165.0
	sigma	3.545	0.138	3.286	3.831	1.00	10301.1	11615.5
	Intercept	168.602	1.016	166.608	170.651	1.00	1084.8	2213.9
	Lineage	6.839	0.942	4.972	8.668	1.00	2920.0	5318.6
	Season	10.303	1.468	7.398	13.171	1.00	1164.5	2532.2
Flowering time (days)	sd (ID)	7.942	0.489	7.039	8.955	1.00	1647.7	2677.8
	sigma	2.982	0.118	2.763	3.226	1.00	9947.3	10723.6
	Intercept	174.790	0.953	172.908	176.642	1.00	989.8	1976.6
	Lineage	5.832	0.792	4.280	7.382	1.00	3159.6	5467.6
	Season	10.407	1.329	7.756	12.932	1.00	813.0	1650.8
Spike length (cm)	sd (ID)	1.331	0.095	1.159	1.528	1.00	4339.5	8480.6
	sigma	0.919	0.037	0.851	0.994	1.00	12323.1	12229.9
	Intercept	9.302	0.173	8.963	9.637	1.00	3496.9	6798.8
	Lineage	1.469	0.202	1.079	1.869	1.00	5791.2	8841.5
	Season	1.286	0.240	0.820	1.755	1.00	3052.0	5319.7
Number of spikelets	sd (ID)	3.763	0.272	3.267	4.328	1.00	4128.7	8283.6
	sigma	2.628	0.106	2.429	2.847	1.00	11100.0	11604.8
	Intercept	23.277	0.482	22.318	24.228	1.00	3855.8	6516.7
	Lineage	7.365	0.581	6.219	8.498	1.00	5876.8	9433.8
	Season	6.738	0.669	5.419	8.042	1.00	3395.1	6453.6
Spikelet length (cm)	sd (ID)	0.070	0.005	0.061	0.081	1.00	4846.5	8628.8
	sigma	0.052	0.002	0.048	0.056	1.00	11214.5	11148.5
	Intercept	1.195	0.009	1.177	1.213	1.00	4579.0	7320.8
	Lineage	-0.073	0.011	-0.094	-0.051	1.00	6338.7	9824.4
	Season	0.052	0.013	0.027	0.077	1.00	4412.4	7411.3
Spikelet width (cm)	sd (ID)	0.019	0.001	0.016	0.021	1.00	5098.4	7798.2
	sigma	0.014	0.001	0.013	0.016	1.00	13101.5	12029.1
	Intercept	0.271	0.002	0.266	0.276	1.00	7067.1	10480.9
	Lineage	-0.040	0.003	-0.045	-0.034	1.00	12107.3	13125.6
	Season	0.031	0.003	0.025	0.038	1.00	6364.6	9479.3
Plant height (cm)	sd (ID)	12.357	0.887	10.746	14.190	1.00	4899.8	8905.2
	sigma	9.061	0.366	8.384	9.809	1.00	13000.7	12434.4
	Intercept	80.071	1.604	76.939	83.201	1.00	4648.6	8082.2
	Lineage	0.569	2.003	-3.291	4.553	1.00	6828.7	9821.2
	Season	19.150	2.266	14.665	23.546	1.00	4154.6	7086.0

S5 Table. (Continued.)

Traits	Effects	Estimate	Est.Error	l-95% CI	u-95% CI	Rhat	Bulk ESS	Tail ESS
1st Internode length (cm)	sd (ID)	6.645	0.487	5.756	7.643	1.00	4443.6	7523.2
	sigma	4.689	0.193	4.328	5.078	1.00	11276.2	12075.6
	Intercept	30.453	0.872	28.722	32.179	1.00	3966.5	6508.7
	Lineage	0.151	1.054	-1.935	2.222	1.00	5448.7	8449.7
	Season	9.050	1.180	6.675	11.355	1.00	3659.4	6309.2
2nd Internode length (cm)	sd (ID)	2.781	0.202	2.414	3.202	1.00	4520.6	8101.8
	sigma	2.030	0.082	1.879	2.199	1.00	12518.8	11577.9
	Intercept	16.046	0.371	15.310	16.781	1.00	3856.3	6388.7
	Lineage	0.520	0.430	-0.313	1.371	1.00	5848.5	8928.5
	Season	2.687	0.512	1.686	3.711	1.00	3561.4	5628.6
3rd Internode length (cm)	sd (ID)	1.721	0.128	1.485	1.991	1.00	4411.4	7878.9
	sigma	1.374	0.055	1.270	1.488	1.00	12552.6	11417.4
	Intercept	10.983	0.230	10.528	11.427	1.00	5337.1	8290.8
	Lineage	0.280	0.278	-0.261	0.823	1.00	7706.3	10023.7
	Season	2.153	0.316	1.536	2.772	1.00	4698.6	7714.1
4th Internode length (cm)	sd (ID)	1.743	0.127	1.509	2.003	1.00	5077.9	8585.4
	sigma	1.329	0.054	1.231	1.439	1.00	12856.1	12486.7
	Intercept	8.067	0.231	7.614	8.526	1.00	5118.5	8076.4
	Lineage	0.385	0.278	-0.173	0.926	1.00	6879.2	9699.7
	Season	1.969	0.317	1.348	2.591	1.00	4521.2	7571.1
5th Internode length (cm)	sd (ID)	2.409	0.206	2.016	2.826	1.00	6087.8	9708.5
	sigma	2.577	0.108	2.375	2.802	1.00	13113.4	12194.1
	Intercept	4.673	0.348	3.996	5.368	1.00	8082.1	11189.6
	Lineage	0.970	0.476	0.037	1.884	1.00	9601.1	11651.3
	Season	1.729	0.475	0.780	2.666	1.00	7729.1	9416.8
Flag leaf length (cm)	sd (ID)	2.514	0.200	2.146	2.935	1.00	4547.6	8323.2
	sigma	2.106	0.087	1.942	2.283	1.00	10350.0	11033.9
	Intercept	6.258	0.342	5.588	6.933	1.00	4973.1	8546.3
	Lineage	1.940	0.442	1.080	2.813	1.00	5778.0	8339.9
	Season	5.651	0.466	4.757	6.566	1.00	4772.7	8068.2

S5 Table. (Continued)

Traits	Effects	Estimate	Est.Error	l-95% CI	u-95% CI	Rhat	Bulk ESS	Tail ESS
Flag leaf width (cm)	sd (ID)	0.096	0.008	0.082	0.111	1.00	5237.2	9707.6
	sigma	0.087	0.004	0.080	0.094	1.00	11862.4	12283.2
	Intercept	0.508	0.013	0.482	0.533	1.00	6979.7	10325.7
	Lineage	0.007	0.017	-0.025	0.041	1.00	8172.8	10702.9
	Season	0.245	0.018	0.210	0.281	1.00	5912.4	8868.8
Stem width (cm)	sd (ID)	0.016	0.001	0.014	0.019	1.00	4115.4	6079.5
	sigma	0.015	0.001	0.013	0.016	1.00	8316.5	11677.7
	Intercept	0.119	0.002	0.114	0.123	1.00	5659.2	7566.2
	Lineage	-0.003	0.003	-0.009	0.003	1.00	6272.9	8728.7
	Season	0.035	0.003	0.028	0.041	1.00	5436.8	7683.8
Top awn length (cm)	sd (ID)	2.112	0.141	1.854	2.405	1.00	2502.2	4973.3
	sigma	1.107	0.045	1.025	1.200	1.00	9955.0	11024.6
	Intercept	6.564	0.259	6.051	7.082	1.00	1805.2	3358.5
	Lineage	-0.784	0.268	-1.309	-0.256	1.00	3912.7	6507.1
	Season	2.242	0.378	1.512	2.996	1.00	1664.8	3135.5
Middle awn length (cm)	sd (ID)	2.307	0.152	2.029	2.627	1.00	1928.7	3750.4
	sigma	1.050	0.043	0.971	1.138	1.00	8550.4	10337.2
	Intercept	10.310	0.278	9.746	10.855	1.00	1233.6	2300.3
	Lineage	-0.144	0.275	-0.690	0.398	1.00	3147.3	5840.8
	Season	-0.604	0.395	-1.381	0.168	1.00	1124.6	2153.5
Bottom awn length (cm)	sd (ID)	1.708	0.118	1.487	1.951	1.00	4545.0	7201.9
	sigma	1.196	0.047	1.108	1.293	1.00	13605.0	12521.3
	Intercept	9.392	0.221	8.955	9.821	1.00	3560.5	7046.3
	Lineage	-1.427	0.254	-1.931	-0.928	1.00	5736.2	8624.7
	Season	-5.084	0.311	-5.688	-4.467	1.00	3244.1	6250.0
