# Supplementary material for: Phenotypic effects of Am genomes in nascent synthetic hexaploids derived from interspecific crosses between durum and wild einkorn wheat
Source: PLoS One. 2023 Apr 27;18(4):e0284408. doi: 10.1371/journal.pone.0284408 (PMC10138484; doi:10.1371/journal.pone.0284408)
Supplement: S7 Table — (PDF) [file pone.0284408.s015.pdf]

S7Table. Summary of posterior means of the fixed coefficients for Bayesian GLMM for the traits of the AABBA^{m}A^{m} synthetic hexaploids.

Traits	Effects	Estimate	Est.Error	l-95% CI	u-95% CI	Rhat	Bulk ESS	Tail ESS
Heading time (days)	sd (ID)	3.969	0.179	3.636	4.334	1.00	2682.3	4959.3
	sigma	2.221	0.063	2.102	2.349	1.00	9484.2	10837.7
	Intercept	154.967	0.363	154.263	155.670	1.00	1533.5	3252.9
	Lineage	2.094	0.494	1.148	3.058	1.00	1727.8	3780.4
	Season	7.097	0.468	6.165	7.999	1.00	1608.9	3413.7
Flowering time (days)	sd (ID)	3.879	0.166	3.569	4.216	1.00	2099.5	4618.6
	sigma	1.864	0.053	1.765	1.971	1.00	9804.4	11257.9
	Intercept	159.521	0.347	158.834	160.205	1.01	1109.5	2241.6
	Lineage	2.250	0.475	1.333	3.171	1.00	1305.7	2738.2
	Season	7.955	0.450	7.061	8.845	1.01	1056.2	2432.0
Spike length (cm)	sd (ID)	1.438	0.070	1.307	1.581	1.00	4264.3	7750.9
	sigma	1.093	0.031	1.034	1.157	1.00	10643.4	11073.6
	Intercept	11.569	0.137	11.301	11.838	1.00	3810.2	6481.4
	Lineage	-0.470	0.186	-0.836	-0.108	1.00	3871.7	6732.1
	Season	0.334	0.177	-0.014	0.679	1.00	3677.7	6018.1
Number of spikelets	sd (ID)	3.144	0.149	2.865	3.448	1.00	4252.0	7622.0
	sigma	2.200	0.064	2.078	2.331	1.00	10381.2	11422.1
	Intercept	23.846	0.298	23.254	24.431	1.00	2605.1	4555.9
	Lineage	1.173	0.417	0.359	1.995	1.00	2927.1	5559.2
	Season	1.164	0.378	0.427	1.910	1.00	2927.1	5557.5
Spikelet length (cm)	sd (ID)	0.073	0.004	0.065	0.081	1.00	5212.3	8895.6
	sigma	0.073	0.002	0.069	0.077	1.00	11370.5	11670.6
	Intercept	1.689	0.007	1.674	1.703	1.00	6216.9	9533.4
	Lineage	-0.079	0.010	-0.099	-0.060	1.00	6419.6	9491.9
	Season	-0.044	0.009	-0.063	-0.026	1.00	5809.1	8321.1
Spikelet width (cm)	sd (ID)	0.038	0.002	0.034	0.042	1.00	4902.7	7715.7
	sigma	0.035	0.001	0.034	0.037	1.00	8599.4	10865.0
	Intercept	0.555	0.004	0.547	0.562	1.00	4602.5	7149.6
	Lineage	-0.035	0.005	-0.045	-0.025	1.00	4366.4	7692.3
	Season	0.097	0.005	0.088	0.107	1.00	4368.9	7496.7
Plant height (cm)	sd (ID)	13.360	0.629	12.162	14.635	1.00	3525.1	6987.8
	sigma	9.204	0.264	8.698	9.740	1.00	9916.3	11312.4
	Intercept	140.341	1.269	137.863	142.843	1.00	2729.6	5082.9
	Lineage	11.896	1.731	8.531	15.298	1.00	2750.5	5205.5
	Season	-18.434	1.622	-21.587	-15.203	1.00	2612.0	5148.2

S6 Table. (Continued.)

Traits	Effects	Estimate	Est.Error	l-95% CI	u-95% CI	Rhat	Bulk ESS	Tail ESS
1st Internode length (cm)	sd (ID)	9.492	0.419	8.711	10.355	1.00	2566.9	4763.4
	sigma	5.070	0.144	4.802	5.366	1.00	10955.9	11468.0
	Intercept	53.614	0.868	51.930	55.342	1.00	1501.5	2951.9
	Lineage	6.063	1.177	3.721	8.338	1.00	1744.6	3483.7
	Season	-9.759	1.110	-11.987	-7.595	1.00	1604.7	3334.6
2nd Internode length (cm)	sd (ID)	2.485	0.126	2.251	2.743	1.00	4944.8	8455.0
	sigma	2.093	0.060	1.978	2.216	1.00	11456.8	11364.5
	Intercept	24.820	0.239	24.351	25.287	1.00	4159.8	7018.5
	Lineage	1.790	0.334	1.135	2.432	1.00	4544.4	8049.6
	Season	-8.324	0.309	-8.930	-7.715	1.00	3926.2	6681.7
3rd Internode length (cm)	sd (ID)	2.537	0.111	2.329	2.765	1.00	2575.7	5486.9
	sigma	1.417	0.040	1.340	1.498	1.00	9906.6	10463.6
	Intercept	20.426	0.228	19.983	20.877	1.00	1666.6	3752.3
	Lineage	0.439	0.323	-0.203	1.067	1.00	1688.9	3537.2
	Season	-5.035	0.291	-5.601	-4.467	1.00	1706.3	4018.4
4th Internode length (cm)	sd (ID)	2.112	0.099	1.931	2.316	1.00	3737.6	6199.4
	sigma	1.431	0.041	1.354	1.515	1.00	10929.5	10676.5
	Intercept	17.678	0.197	17.283	18.057	1.00	2742.1	4859.2
	Lineage	1.464	0.276	0.923	2.008	1.00	2921.0	5570.8
	Season	-3.016	0.255	-3.519	-2.514	1.00	2743.9	5122.4
5th Internode length (cm)	sd (ID)	5.616	0.263	5.125	6.153	1.00	4743.0	7815.0
	sigma	4.073	0.115	3.853	4.306	1.00	11702.8	11519.0
	Intercept	12.109	0.534	11.065	13.155	1.00	3244.3	5604.8
	Lineage	2.730	0.734	1.292	4.168	1.00	3221.6	6317.8
	Season	7.270	0.685	5.927	8.621	1.00	3277.5	5649.8
Flag leaf length (cm)	sd (ID)	3.508	0.170	3.186	3.851	1.00	4895.3	8377.2
	sigma	2.740	0.078	2.590	2.899	1.00	11372.5	12323.9
	Intercept	25.520	0.337	24.865	26.174	1.00	3739.7	7105.0
	Lineage	-1.080	0.459	-1.982	-0.192	1.00	4032.6	7786.5
	Season	-1.788	0.433	-2.637	-0.947	1.00	4073.4	7209.8

S6 Table. (Continued)

Traits	Effects	Estimate	Est.Error	l-95% CI	u-95% CI	Rhat	Bulk ESS	Tail ESS
Flag leaf width (cm)	sd (ID)	0.135	0.007	0.123	0.149	1.00	5265.8	8009.3
	sigma	0.115	0.003	0.108	0.121	1.00	10164.5	10709.8
	Intercept	1.401	0.013	1.375	1.427	1.00	4670.1	7794.7
	Lineage	-0.111	0.018	-0.146	-0.076	1.00	4565.9	7809.2
	Season	0.101	0.017	0.067	0.134	1.00	4708.2	8143.2
Stem width (cm)	sd (ID)	0.025	0.001	0.023	0.028	1.00	4900.0	7780.5
	sigma	0.026	0.001	0.024	0.027	1.00	9624.6	11232.7
	Intercept	0.240	0.003	0.235	0.245	1.00	4858.0	7522.5
	Lineage	0.002	0.004	-0.005	0.009	1.00	4408.1	7667.3
	Season	0.018	0.003	0.012	0.025	1.00	4674.9	7657.7
Top awn length (cm)	sd (ID)	1.335	0.071	1.199	1.476	1.00	5398.5	8861.2
	sigma	1.252	0.036	1.185	1.323	1.00	12085.7	11368.4
	Intercept	10.372	0.132	10.108	10.626	1.00	5233.8	7347.4
	Lineage	-1.265	0.184	-1.627	-0.900	1.00	5361.2	8534.5
	Season	-0.266	0.170	-0.601	0.072	1.00	5342.2	8348.8
Middle awn length (cm)	sd (ID)	1.518	0.070	1.384	1.660	1.00	3605.5	6331.3
	sigma	0.983	0.028	0.930	1.040	1.00	10429.6	10891.5
	Intercept	14.976	0.138	14.707	15.247	1.00	2166.6	4228.8
	Lineage	-1.192	0.195	-1.580	-0.809	1.00	1791.1	4381.5
	Season	-1.321	0.177	-1.670	-0.975	1.00	2299.8	4529.9
Bottom awn length (cm)	sd (ID)	1.322	0.075	1.179	1.472	1.00	5414.0	8761.1
	sigma	1.404	0.040	1.330	1.484	1.00	10850.3	11828.1
	Intercept	12.007	0.136	11.741	12.271	1.00	6759.8	9457.5
	Lineage	-1.203	0.189	-1.572	-0.835	1.00	7024.0	9887.3
	Season	-4.668	0.176	-5.012	-4.320	1.00	6728.1	9397.8
