# Supplementary material for: Phenotypic effects of Am genomes in nascent synthetic hexaploids derived from interspecific crosses between durum and wild einkorn wheat
Source: PLoS One. 2023 Apr 27;18(4):e0284408. doi: 10.1371/journal.pone.0284408 (PMC10138484; doi:10.1371/journal.pone.0284408)
Supplement: S8 Table — (PDF) [file pone.0284408.s016.pdf]

S8 Table. Summary of posterior means of the fixed coefficients for Bayesian GLM without the parameter of thermal time after anthesis for the grain traits of *Triticum monococcum* ssp. *aegilopoides*.

Traits	Effects	Estimate	Est.Error	l-95% CI	u-95% CI	Rhat	Bulk ESS	Tail ESS
Grain length (mm)	sigma	0.679	0.011	0.657	0.702	1.00	18536.9	12184.0
	Intercept	6.829	0.018	6.792	6.864	1.00	16883.1	11930.2
	Lineage	-0.756	0.037	-0.828	-0.682	1.00	14029.9	11862.7
Grain width (mm)	sigma	0.258	0.004	0.250	0.267	1.00	13756.9	11640.9
	Intercept	1.242	0.007	1.229	1.256	1.00	17850.7	12176.7
	Lineage	-0.110	0.014	-0.137	-0.081	1.00	15536.9	11566.9
Grain perimeter length (mm)	sigma	1.568	0.026	1.518	1.619	1.00	17476.3	12240.2
	Intercept	15.655	0.043	15.572	15.739	1.00	14581.6	11389.3
	Lineage	-1.802	0.087	-1.970	-1.631	1.00	15684.3	12168.2
Grain area (mm2)	sigma	1.574	0.026	1.523	1.626	1.00	14907.7	12184.8
	Intercept	6.364	0.042	6.282	6.446	1.00	16689.3	11714.9
	Lineage	-1.155	0.086	-1.322	-0.987	1.00	14336.6	11216.4
Grain circularity	sigma	0.051	0.001	0.049	0.052	1.00	7728.9	8225.1
	Intercept	0.324	0.001	0.321	0.326	1.00	17389.9	12818.7
	Lineage	0.012	0.003	0.006	0.017	1.00	10332.8	10014.3
