# Supplementary material for: Phenotypic effects of Am genomes in nascent synthetic hexaploids derived from interspecific crosses between durum and wild einkorn wheat
Source: PLoS One. 2023 Apr 27;18(4):e0284408. doi: 10.1371/journal.pone.0284408 (PMC10138484; doi:10.1371/journal.pone.0284408)
Supplement: S9 Table — (PDF) [file pone.0284408.s017.pdf]

S9 Table. Summary of posterior means of the fixed coefficients for Bayesian GLM without the parameter of thermal time after anthesis for the grain traits of the synthetic hexaploids.

Traits	Effects	Estimate	Est.Error	l-95% CI	u-95% CI	Rhat	Bulk ESS	Tail ESS
Grain length (mm)	sigma	0.763	0.012	0.740	0.787	1.00	15825.7	11694.6
	Intercept	8.944	0.020	8.904	8.983	1.00	16300.2	12437.0
	Lineage	-0.566	0.038	-0.641	-0.491	1.00	14446.6	10893.2
Grain width (mm)	sigma	0.331	0.005	0.320	0.341	1.00	17582.1	11690.4
	Intercept	2.207	0.009	2.190	2.224	1.00	18841.5	11911.8
	Lineage	-0.301	0.017	-0.333	-0.269	1.00	17524.7	12313.6
Grain perimeter length (mm)	sigma	1.757	0.028	1.703	1.814	1.00	16637.1	12446.7
	Intercept	20.610	0.046	20.518	20.700	1.00	15684.8	11472.7
	Lineage	-1.529	0.088	-1.699	-1.358	1.00	14995.1	11689.2
Grain area (mm2)	sigma	2.646	0.041	2.567	2.730	1.00	15388.3	11310.2
	Intercept	14.428	0.070	14.295	14.566	1.00	14086.0	11563.3
	Lineage	-2.499	0.132	-2.758	-2.237	1.00	15917.0	12146.8
Grain circularity	sigma	0.046	0.001	0.044	0.047	1.00	5701.4	6637.6
	Intercept	0.424	0.001	0.422	0.427	1.00	16537.9	13384.7
	Lineage	-0.014	0.002	-0.018	-0.009	1.00	10757.9	9371.1
