# Supplementary material for: Phenotypic effects of Am genomes in nascent synthetic hexaploids derived from interspecific crosses between durum and wild einkorn wheat
Source: PLoS One. 2023 Apr 27;18(4):e0284408. doi: 10.1371/journal.pone.0284408 (PMC10138484; doi:10.1371/journal.pone.0284408)
Supplement: S10 Table — (PDF) [file pone.0284408.s018.pdf]

S10 Table. Summary of posterior means of the fixed coefficients for Bayesian GLMM with the parameter of thermal time after anthesis for the grain traits of *Triticum monococcum* ssp. *aegilopoides*.

Traits	Effects	Estimate	Est.Error	l-95% CI	u-95% CI	Rhat	Bulk ESS	Tail ESS
Grain length (mm)	sd (T600C))	0.605	0.110	0.433	0.855	1.00	1749	3255
	sigma	0.543	0.009	0.526	0.562	1.00	8204	8548
	Intercept	6.707	0.138	6.435	6.993	1.00	1451	2101
	Lineage	-0.428	0.055	-0.536	-0.321	1.00	6210	7322
Grain width (mm)	sd (T600C)	0.189	0.035	0.135	0.270	1.00	2027	3507
	sigma	0.206	0.003	0.200	0.213	1.00	9664	9978
	Intercept	1.188	0.044	1.099	1.276	1.00	1821	2517
	Lineage	0.049	0.021	0.008	0.090	1.00	7093	8147
Grain perimeter length (mm)	sd (T600C)	1.419	0.255	1.021	1.998	1.00	2004	3588
	sigma	1.220	0.021	1.180	1.260	1.00	7749	7775
	Intercept	15.340	0.327	14.680	15.979	1.00	1349	2005
	Lineage	-0.954	0.126	-1.199	-0.706	1.00	6435	7189
Grain area (mm^2)	sd (T600C)	1.316	0.227	0.962	1.840	1.00	1929	4089
	sigma	1.196	0.020	1.158	1.236	1.00	8103	8920
	Intercept	6.023	0.297	5.431	6.614	1.00	1322	2337
	Lineage	-0.165	0.121	-0.401	0.071	1.00	7037	8024
Grain circularity	sd (T600C)	0.026	0.005	0.018	0.037	1.00	2621	5047
	sigma	0.045	0.001	0.044	0.047	1.00	12520	11201
	Intercept	0.319	0.006	0.307	0.331	1.00	2123	3311
	Lineage	0.028	0.004	0.019	0.036	1.00	8366	10154
