# Supplementary material for: Phenotypic effects of Am genomes in nascent synthetic hexaploids derived from interspecific crosses between durum and wild einkorn wheat
Source: PLoS One. 2023 Apr 27;18(4):e0284408. doi: 10.1371/journal.pone.0284408 (PMC10138484; doi:10.1371/journal.pone.0284408)
Supplement: S11 Table — (PDF) [file pone.0284408.s019.pdf]

S11 Table. Summary of posterior means of the fixed coefficients for Bayesian GLMM with the parameter of thermal time after anthesis for the grain traits of the synthetic hexaploids.

Traits	Effects	Estimate	Est.Error	l-95% CI	u-95% CI	Rhat	Bulk ESS	Tail ESS
Grain length (mm)	sd (T600C))	0.492	0.132	0.306	0.823	1.00	3288.3	5232.2
	sigma	0.692	0.011	0.670	0.714	1.00	10766.7	9493.9
	Intercept	8.656	0.158	8.345	8.975	1.00	2627.3	3679.6
	Lineage	-0.365	0.045	-0.453	-0.276	1.00	11334.6	10321.6
Grain width (mm)	sd (T600C)	0.175	0.048	0.107	0.294	1.00	3001.3	5101.6
	sigma	0.310	0.005	0.301	0.320	1.00	11781.4	10400.9
	Intercept	2.115	0.056	1.999	2.230	1.00	3167.3	4188.2
	Lineage	-0.222	0.020	-0.261	-0.183	1.00	10887.2	10435.5
Grain perimeter length (mm)	sd (T600C)	1.105	0.291	0.694	1.810	1.00	3184.4	5604.7
	sigma	1.596	0.025	1.547	1.645	1.00	11490.1	9942.7
	Intercept	19.941	0.345	19.277	20.629	1.00	3081.4	4323.4
	Lineage	-1.046	0.102	-1.247	-0.848	1.00	10426.9	9685.4
Grain area (mm^2)	sd (T600C)	1.456	0.378	0.919	2.384	1.00	2961.2	5404.5
	sigma	2.427	0.039	2.351	2.505	1.00	11058.9	9714.0
	Intercept	13.407	0.457	12.497	14.300	1.00	3257.3	4935.1
	Lineage	-1.672	0.160	-1.985	-1.359	1.00	11225.7	10134.0
Grain circularity	sd (T600C)	0.022	0.006	0.013	0.038	1.00	3068.9	4474.1
	sigma	0.043	0.001	0.042	0.045	1.00	10801.9	9980.8
	Intercept	0.419	0.007	0.406	0.433	1.00	3162.0	4657.5
	Lineage	-0.007	0.003	-0.013	-0.002	1.00	14019.3	11574.4
