# Supplementary material for: Phenotypic effects of Am genomes in nascent synthetic hexaploids derived from interspecific crosses between durum and wild einkorn wheat
Source: PLoS One. 2023 Apr 27;18(4):e0284408. doi: 10.1371/journal.pone.0284408 (PMC10138484; doi:10.1371/journal.pone.0284408)
Supplement: S12 Table — (PDF) [file pone.0284408.s020.pdf]

S12 Table. Results of the model comparisons based on WAIC.

Traits	*Triticum monococcum* ssp. *aegilopoides*		Synthetic hexaploids	
	Difference of ELPD (SE) Model B - Model C	Supported model	Difference of ELPD (SE) Model B - Model C	Supported model
Grain length	-395.3 (27.1)	Model C	-190.7 (20.9)	Model C
Grain width	-398.6 (25.5)	Model C	-124.2 (14.2)	Model C
Grain perimeter length	-448.1 (28.3)	Model C	-189.2 (20.1)	Model C
Grain area	-489.7 (27.6)	Model C	-168.8 (16.3)	Model C
Grain circularity	-201.4 (19.2)	Model C	-97.7 (13.9)	Model C

Model B is GLM. Model C is GLMM. Model C was a model incorporated 600°C thermal days into Model B as random effect.
