# Supplementary material for: Phenotypic effects of Am genomes in nascent synthetic hexaploids derived from interspecific crosses between durum and wild einkorn wheat
Source: PLoS One. 2023 Apr 27;18(4):e0284408. doi: 10.1371/journal.pone.0284408 (PMC10138484; doi:10.1371/journal.pone.0284408)
Supplement: S13 Table — (PDF) [file pone.0284408.s021.pdf]

S13 Table. Summary of posterior means of the fixed coefficients for Bayesian GLMM for the traits between the synthetic hexaploids of $AABBA^mA^m$ (ABA^m), AABBAA (ABA), and AABBDD (ABD).

Traits	Effects	Estimate	Est.Error	l-95% CI	u-95% CI	Rhat	Bulk ESS	Tail ESS
Heading time (days)	sd (ID)	4.048	0.166	3.733	4.387	1.00	3595.6	5196.9
	sigma	2.260	0.059	2.148	2.378	1.00	11504.7	12164.8
	Intercept	153.103	1.511	150.066	156.062	1.00	3717.2	6484.1
	ABA	-1.811	2.148	-5.958	2.507	1.00	4349.1	6432.3
	ABAm	2.708	1.516	-0.287	5.695	1.00	3735.2	6534.2
	ABD	-4.990	1.623	-8.178	-1.791	1.00	3485.7	6275.0
	Season	6.715	0.438	5.870	7.572	1.00	2836.7	5373.5
Flowering time (days)	sd (ID)	3.932	0.157	3.638	4.251	1.00	3823.1	6261.4
	sigma	1.898	0.051	1.803	1.999	1.00	12524.3	12632.9
	Intercept	156.143	1.461	153.355	159.053	1.00	5034.4	7639.2
	ABA	-0.379	2.013	-4.338	3.547	1.00	5241.1	8074.0
	ABAm	4.452	1.460	1.549	7.291	1.00	5102.3	7897.6
	ABD	-2.078	1.600	-5.271	1.029	1.00	4667.5	7371.5
	Season	7.245	0.414	6.421	8.065	1.00	3126.5	5752.4
Spike length (cm)	sd (ID)	1.418	0.065	1.294	1.550	1.00	5055.1	8721.3
	sigma	1.132	0.030	1.076	1.193	1.00	11600.5	12747.4
	Intercept	8.330	0.558	7.274	9.445	1.00	4891.3	7809.9
	ABA	1.779	0.784	0.212	3.283	1.00	4717.6	8055.4
	ABAm	3.044	0.561	1.927	4.108	1.00	4900.0	7960.9
	ABD	5.823	0.608	4.603	6.993	1.00	4296.4	7632.9
	Season	0.430	0.165	0.102	0.753	1.00	4348.0	7405.7
Number of spikelets	sd (ID)	3.010	0.137	2.752	3.287	1.00	4456.6	8323.7
	sigma	2.189	0.059	2.079	2.308	1.00	11525.9	12691.2
	Intercept	21.086	1.152	18.809	23.363	1.00	3762.0	6590.0
	ABA	2.159	1.635	-1.098	5.265	1.00	4349.5	7268.2
	ABAm	3.079	1.160	0.794	5.349	1.00	3779.8	6904.9
	ABD	-1.196	1.291	-3.746	1.374	1.00	3542.0	6556.0
	Season	1.261	0.343	0.592	1.933	1.00	3509.6	5895.6
Spikelet length (cm)	sd (ID)	0.089	0.004	0.081	0.097	1.00	5445.6	9661.6
	sigma	0.075	0.002	0.071	0.079	1.00	13918.2	13066.1
	Intercept	1.487	0.035	1.418	1.557	1.00	6138.2	8967.9
	ABA	0.083	0.049	-0.014	0.181	1.00	6298.2	9679.0
	ABAm	0.164	0.035	0.094	0.234	1.00	6171.2	9059.5
	ABD	-0.089	0.039	-0.166	-0.010	1.00	6156.1	9167.2
	Season	-0.019	0.010	-0.039	0.002	1.00	6024.2	8929.2

S12 Table. (Continued)

Traits	Effects	Estimate	Est.Error	l-95% CI	u-95% CI	Rhat	Bulk ESS	Tail ESS
Spikelet width (cm)	sd (ID)	0.044	0.002	0.040	0.048	1.00	5510.8	9489.2
	sigma	0.036	0.001	0.035	0.038	1.00	12229.0	12508.4
	Intercept	0.572	0.017	0.538	0.606	1.00	4727.6	8708.1
	ABA	-0.068	0.024	-0.115	-0.021	1.00	5602.2	8712.6
	ABAm	-0.024	0.017	-0.058	0.009	1.00	4699.6	7027.8
	ABD	0.024	0.019	-0.013	0.062	1.00	4790.9	8357.6
	Season	0.090	0.005	0.080	0.100	1.00	4781.2	8491.0
Plant height (cm)	sd (ID)	14.315	0.631	13.125	15.571	1.00	4532.6	8450.7
	sigma	10.068	0.269	9.553	10.605	1.00	11989.8	13151.5
	Intercept	135.950	5.497	125.124	146.656	1.00	3955.5	7137.3
	ABA	-17.899	7.638	-32.686	-2.911	1.00	4144.8	7093.1
	ABAm	6.370	5.507	-4.303	17.223	1.00	3901.0	6982.3
	ABD	-1.561	6.123	-13.605	10.335	1.00	3669.3	7015.1
	Season	-14.912	1.599	-18.089	-11.821	1.00	3251.9	5934.7
1st Internode length (cm)	sd (ID)	9.363	0.385	8.644	10.152	1.00	2706.9	5442.6
	sigma	5.095	0.136	4.838	5.370	1.00	12034.6	11585.9
	Intercept	50.807	3.523	43.938	57.819	1.00	3579.6	5773.3
	ABA	-6.090	4.945	-15.770	3.684	1.00	3988.1	6319.0
	ABAm	4.509	3.537	-2.538	11.464	1.00	3539.9	5782.7
	ABD	-11.464	3.910	-19.223	-3.723	1.00	3201.7	5906.4
	Season	-9.350	1.031	-11.369	-7.334	1.00	2424.5	3944.0
2nd Internode length (cm)	sd (ID)	2.674	0.126	2.435	2.930	1.00	5336.7	9247.2
	sigma	2.206	0.059	2.096	2.326	1.00	13823.3	12707.6
	Intercept	21.589	1.061	19.492	23.602	1.00	4880.4	8002.7
	ABA	0.565	1.484	-2.366	3.482	1.00	5515.7	8846.4
	ABAm	3.423	1.062	1.386	5.519	1.00	4961.1	8072.3
	ABD	1.455	1.183	-0.823	3.782	1.00	4892.3	7605.8
	Season	-7.579	0.309	-8.183	-6.973	1.00	4367.8	6896.9
3rd Internode length (cm)	sd (ID)	2.671	0.112	2.460	2.897	1.00	3218.0	6403.2
	sigma	1.545	0.041	1.468	1.629	1.00	11619.0	11534.2
	Intercept	17.307	1.004	15.326	19.268	1.00	2662.5	4958.9
	ABA	0.339	1.408	-2.411	3.124	1.00	3372.2	5639.1
	ABAm	2.842	1.004	0.881	4.835	1.00	2797.7	5143.3
	ABD	2.533	1.120	0.337	4.730	1.00	2543.9	5008.5
	Season	-4.195	0.296	-4.769	-3.613	1.00	1885.4	3693.8

S12 Table. (Continued)

Traits	Effects	Estimate	Est.Error	l-95% CI	u-95% CI	Rhat	Bulk ESS	Tail ESS
4th Internode length (cm)	sd (ID)	2.232	0.099	2.046	2.433	1.00	5052.0	8620.3
	sigma	1.640	0.043	1.558	1.728	1.00	13806.2	12235.0
	Intercept	16.202	0.864	14.510	17.922	1.00	3825.4	7333.8
	ABA	0.205	1.220	-2.195	2.574	1.00	4389.2	7930.3
	ABAm	1.628	0.862	-0.083	3.312	1.00	3853.7	7362.5
	ABD	2.355	0.961	0.437	4.201	1.00	3722.4	6939.5
	Season	-2.392	0.253	-2.896	-1.909	1.00	4416.9	7739.0
5th Internode length (cm)	sd (ID)	5.576	0.256	5.094	6.093	1.00	5236.6	9681.8
	sigma	4.590	0.123	4.356	4.841	1.00	12957.2	12568.7
	Intercept	22.013	2.199	17.672	26.289	1.00	5073.3	7753.4
	ABA	-14.853	3.018	-20.777	-8.972	1.00	4998.8	7904.7
	ABAm	-9.379	2.202	-13.623	-5.030	1.00	5116.0	7387.5
	ABD	-2.683	2.452	-7.553	2.152	1.00	4889.3	7297.8
	Season	7.942	0.640	6.693	9.194	1.00	4884.2	8454.0
Flag leaf length (cm)	sd (ID)	3.495	0.161	3.192	3.819	1.00	5355.0	8439.1
	sigma	2.821	0.075	2.678	2.973	1.00	12960.6	12518.1
	Intercept	22.048	1.382	19.366	24.766	1.00	4862.4	7899.7
	ABA	-0.422	1.942	-4.248	3.372	1.00	5076.5	8252.5
	ABAm	2.843	1.383	0.106	5.514	1.00	4986.4	8175.5
	ABD	3.235	1.522	0.234	6.216	1.00	5003.3	8338.2
	Season	-1.199	0.403	-1.994	-0.419	1.00	4240.0	7053.9
Flag leaf width (cm)	sd (ID)	0.137	0.007	0.124	0.150	1.00	5645.8	9242.2
	sigma	0.123	0.003	0.117	0.130	1.00	12969.4	12625.4
	Intercept	1.371	0.055	1.262	1.477	1.00	4904.5	7888.2
	ABA	-0.156	0.076	-0.304	-0.006	1.00	5720.1	8913.3
	ABAm	-0.004	0.055	-0.112	0.105	1.00	4883.7	8142.6
	ABD	0.044	0.060	-0.073	0.162	1.00	4785.2	8037.1
	Season	0.098	0.016	0.067	0.129	1.00	6094.1	9882.2
Stem width (cm)	sd (ID)	0.025	0.001	0.022	0.027	1.00	6400.8	9737.9
	sigma	0.027	0.001	0.025	0.028	1.00	12882.4	12677.5
	Intercept	0.235	0.010	0.215	0.255	1.00	8856.5	11343.3
	ABA	-0.038	0.015	-0.067	-0.009	1.00	9775.7	12016.7
	ABAm	0.006	0.010	-0.014	0.027	1.00	8779.7	11442.9
	ABD	-0.005	0.011	-0.028	0.017	1.00	9015.1	11239.7
	Season	0.016	0.003	0.010	0.022	1.00	11045.5	11603.3

S12 Table. (Continued)

Traits	Effects	Estimate	Est.Error	l-95% CI	u-95% CI	Rhat	Bulk ESS	Tail ESS
Top awn length (cm)	sd (ID)	1.415	0.067	1.286	1.552	1.00	6051.9	8888.6
	sigma	1.208	0.032	1.147	1.273	1.00	13339.8	13120.6
	Intercept	10.095	0.563	8.992	11.205	1.00	5802.5	9146.2
	ABA	-4.202	0.791	-5.733	-2.642	1.00	6411.7	9417.4
	ABAm	-0.159	0.564	-1.270	0.942	1.00	5723.2	9108.7
	ABD	-2.686	0.617	-3.871	-1.481	1.00	5707.0	9249.0
	Season	-0.185	0.165	-0.508	0.142	1.00	5458.5	8821.4
Middle awn length (cm)	sd (ID)	1.570	0.066	1.444	1.707	1.00	3062.7	5465.1
	sigma	0.969	0.026	0.920	1.021	1.00	10317.5	11431.3
	Intercept	13.640	0.590	12.481	14.801	1.00	2797.6	5141.0
	ABA	-4.395	0.824	-5.998	-2.752	1.00	2956.9	5156.5
	ABAm	0.865	0.590	-0.291	2.027	1.00	2784.2	5094.5
	ABD	-5.334	0.640	-6.584	-4.076	1.00	2784.1	5264.9
	Season	-1.126	0.172	-1.463	-0.784	1.00	1872.2	3846.6
Bottom awn length (cm)	sd (ID)	1.542	0.073	1.403	1.688	1.00	5552.1	10086.8
	sigma	1.355	0.036	1.286	1.428	1.00	14658.9	12552.4
	Intercept	11.146	0.613	9.934	12.345	1.00	6264.8	8860.5
	ABA	-3.194	0.850	-4.862	-1.533	1.00	6318.4	9578.9
	ABAm	0.223	0.615	-0.982	1.434	1.00	6204.7	9088.8
	ABD	-4.721	0.671	-6.034	-3.398	1.00	5992.3	8703.5
	Season	-4.140	0.180	-4.493	-3.786	1.00	5930.3	9106.9
