# Supplementary material for: Phenotypic effects of Am genomes in nascent synthetic hexaploids derived from interspecific crosses between durum and wild einkorn wheat
Source: PLoS One. 2023 Apr 27;18(4):e0284408. doi: 10.1371/journal.pone.0284408 (PMC10138484; doi:10.1371/journal.pone.0284408)
Supplement: S14 Table — (PDF) [file pone.0284408.s022.pdf]

S14 Table. Summary of the color of grain surface of Langdon and the synthetic hexaploids with AABBA^{m}A^m (ABA^m), AABBAA (ABA), and AABBDD (ABD) genomes.

accession	Syn	genome	CIE Lab L*	CIE Lab a*	CIE Lab b*
Langdon	-	AB	56.44	8.18	21.75
Ldn/KU-2097	6214	ABD	50.12	10.34	19.89
Ldn/IG 126387	6240	ABD	48.87	10.98	22.02
Ldn/PI 476874	6256	ABD	52.81	10.07	20.32
Ldn/KU-2069	6262	ABD	53.31	9.46	18.99
Ldn/KU-101-2	7100	ABA^m	56.09	7.85	16.82
Ldn/KU-101-3	7101	ABA^m	53.66	7.86	17.55
Ldn/KU-1501	7102	ABA^m	53.73	7.09	16.66
Ldn/KU-1507	7103	ABA^m	54.67	6.67	15.54
Ldn/KU-1516	7104	ABA^m	54.71	6.94	16.01
Ldn/KU-3620	7105	ABA^m	53.11	8.30	18.50
Ldn/KU-3630	7106	ABA^m	55.73	7.22	15.94
Ldn/KU-3646	7107	ABA^m	54.55	7.41	17.04
Ldn/KU-8001	7108	ABA^m	52.00	6.41	16.14
Ldn/KU-8111	7109	ABA^m	53.93	8.33	19.07
Ldn/KU-8116	7110	ABA^m	53.61	6.83	17.02
Ldn/KU-8120	7111	ABA^m	54.03	9.14	19.86
Ldn/KU-8122	7112	ABA^m	54.44	6.11	17.83
Ldn/KU-8125	7113	ABA^m	54.77	8.51	18.77
Ldn/KU-8136	7114	ABA^m	55.70	8.09	19.86
Ldn/KU-8139	7115	ABA^m	55.03	6.44	15.02
Ldn/KU-8143	7116	ABA^m	52.85	6.27	16.06
Ldn/KU-8162	7117	ABA^m	52.76	6.34	15.31
Ldn/KU-8186	7118	ABA^m	54.42	6.98	16.17
Ldn/KU-8201	7119	ABA^m	56.25	6.02	15.25
Ldn/KU-8223	7120	ABA^m	53.92	7.53	17.30
Ldn/KU-8241	7121	ABA^m	52.70	6.18	15.13
Ldn/KU-8266	7122	ABA^m	54.49	6.88	16.10
Ldn/KU-8269	7124	ABA^m	53.52	7.67	17.18
Ldn/KU-8279	7126	ABA^m	55.96	7.21	16.32
Ldn/KU-8287	7127	ABA^m	55.18	7.19	16.32
Ldn/KU-8297	7128	ABA^m	52.48	7.87	17.17
Ldn/KU-8315	7129	ABA^m	54.19	6.98	16.22
Ldn/KU-8322	7130	ABA^m	52.55	7.90	18.35
Ldn/KU-8332	7131	ABA^m	52.30	8.56	18.86
Ldn/KU-8345	7132	ABA^m	54.42	5.98	15.03
Ldn/KU-8404	7134	ABA^m	51.73	6.81	16.19
Ldn/KU-8405	7135	ABA^m	51.67	6.50	15.94
Ldn/KU-8414	7136	ABA^m	50.75	6.84	16.05
Ldn/KU-10603	7137	ABA^m	53.22	7.38	16.53
Ldn/KU-10653	7138	ABA^m	53.84	7.36	16.41
Ldn/KU-10830	7139	ABA^m	52.66	6.79	16.88
Ldn/KU-10859	7140	ABA^m	53.30	7.67	18.00
Ldn/PI427634	7141	ABA^m	52.52	9.01	19.78
Ldn/KU-199-16	7142	ABA	54.22	8.89	18.39
