# Supplementary material for: Phenotypic effects of Am genomes in nascent synthetic hexaploids derived from interspecific crosses between durum and wild einkorn wheat
Source: PLoS One. 2023 Apr 27;18(4):e0284408. doi: 10.1371/journal.pone.0284408 (PMC10138484; doi:10.1371/journal.pone.0284408)
Supplement: S15 Table — (PDF) [file pone.0284408.s023.pdf]

S15 Table. Comparison of phenotypic traits among *Triticum monococcum* ssp. *aegilopoides*, *T. urartu*, *Aegilops tauschii*, and *Ae. umbellulata* that were used as the pollen parents of the synthetic hexaploids.

Traits (units)	Species			
	T. monococcum ssp. *aegilopoides*	*T. urartu*	*Ae. tauschii*	*Ae. umbellulata*
Flowering time (days)	176	154	168	159
Top awn length (cm)	6.42	1.27	3.20	3.12
1st internode length (cm)	31.0	21.5	25.7	16.0
The number of spikelets	31.9	20.7	13.3	4.78
Spikelet length (cm)	1.18	1.09	0.94	0.93–1.23

The data of wild einkorn *T. monococcum* ssp. *aegilopoides* and *T. urartu* in the 2017–2018 seasons in this study were used. The data of *Ae. tauschii* were referred from Takumi et al. 2009. The data of *Ae. umbellulata* were referred from Okada et al. 2020.
